# Residual Malaria Transmission in Select Countries of Asia-Pacific Region: Old Wine in a New Barrel

**DOI:** 10.1093/infdis/jiab004

**Published:** 2021-04-27

**Authors:** Jeffrey Hii, John Hustedt, Michael J Bangs

**Affiliations:** 1 Malaria Consortium Asia, Faculty of Tropical Medicine, Mahidol University, Bangkok, Thailand; 2 College of Public Health, Medical and Veterinary Sciences, James Cook University, Townsville, Queensland, Australia; 3 Forefront, Phnom Penh, Cambodia; 4 Public Health and Malaria Control Department, PT Freeport Indonesia, International SOS, Jl. Kertajasa, Kuala Kencana, Papua, Indonesia; 5 Department of Entomology, Faculty of Agriculture, Kasertart University, Bangkok, Thailand

**Keywords:** Residual malaria transmission, early outdoor mosquito biting, universal or maximal coverage of ITN and IRS, human behavior, exophagy, nighttime activity

## Abstract

**Background:**

Despite substantial reductions in malaria burden and improvement in case management, malaria remains a major public health challenge in the Asia-Pacific region. Residual malaria transmission (RMT) is the fraction of total transmission that persists after achievement of full operational coverage with effective insecticide-treated bed nets (ITNs)/long-lasting insecticidal nets (LLINs) and/or indoor residual spray interventions. There is a critical need to standardize and share best practices for entomological, anthropological, and product development investigative protocols to meet the challenges of RMT and elimination goals.

**Methods:**

A systematic review was conducted to describe when and where RMT is occurring, while specifically targeting ownership and usage of ITN/LLINs, indoor residual spray application, insecticide susceptibility of vectors, and human and vector biting behavior, with a focus on nighttime activities.

**Results:**

Sixty-six publications from 1995 to present met the inclusion criteria for closer review. Associations between local vector control coverage and use with behaviors of human and mosquito vectors varied by locality and circumstance. Consequently, the magnitude of RMT is insufficiently studied and analyzed with sparse estimates of individual exposure in communities, insufficient or incomplete observations of ITN/LLIN use, and the local human population movement into and from high-risk areas.

**Conclusions:**

This review identified significant gaps or deficiencies that require urgent attention, namely, developing standardized procedures and methods to estimate risk exposure beyond the peridomestic setting, analytical approaches to measure key human-vector interactions, and seasonal location-specific agricultural or forest use calendars, and establishing the collection of longitudinal human and vector data close in time and location.

Massive scale up and substantial expansion of time tested interventions contributed to about 48%–75% decline in malaria incidence [1] and 60%–87% in malaria-related mortality [2] during the last fifteen years in the World Health Organization (WHO) South East Asian and Western Pacific regions, respectively. These efforts have been spurred by increased funding to support large-scale and continuous distribution across all countries of insecticide-treated bed nets (ITNs), also commonly termed long-lasting insecticidal nets (LLINs), indoor residual spraying (IRS) in a limited number of countries, and wider availability of affordable and effective artemisinin-based combination therapy to treat malaria augmented with enhanced case detection and surveillance coverage. An estimated 68% of the decrease in infections can be attributed to global ITN distribution, making this the most effective malaria prevention tool currently available [3, 4]. Combined, the core transmission and vector control interventions, ITNs and IRS, account for an estimated three-quarters of clinical malaria cases averted [3].

Despite the contribution of ITNs and IRS to vector control, malaria persists, with a disproportionate impact in the Asia-Pacific region relative to other WHO regions owing to the biological diversity of *Anopheles* species complexes, human behaviors, and the variable impact of vector control. Generally, effectiveness is high initially for community-based transmission scenarios and low in forest-based situations. In 2018, 3 high-burden Western Pacific countries accounted for 98% of 1 980 034 cases (Papua New Guinea [80%], Cambodia [14%], and Solomon Islands [4%]), and 2 countries in the South-East Asian region accounted for 98% of cases (India [85%] and Indonesia [13%]) [5]. 

Most countries are aiming to eliminate malaria by 2020 (China, Malaysia, and Republic of Korea), 2025 (Bhutan and Cambodia), or 2030 (Myanmar, Lao People’s Democratic Republic [PDR], Vietnam, and Thailand). Together with other countries that are reorienting their programs toward elimination phases (Nepal, Democratic People’s Republic of Korea, Vanuatu) or moving toward subnational elimination targets [1, 2], many are also facing challenges with varying levels of local RTM and/or emergent or increased zoonotic (simian) malaria transmission. Residual malaria transmission (RMT), defined by WHO as “persistence of parasite transmission even with good access to and usage of ITNs or well-implemented IRS, as well as in situations where ITN use or IRS are not practical,” represents a critical challenge for malaria control and elimination efforts [9]. 

The urgency of addressing RMT is heightened by the continued transmission of artemisinin-resistant *Plasmodium falciparum* parasites in countries of the Greater Mekong Subregion (GMS) that should they spread beyond the Myanmar-Northeastern India border [10], threaten global malaria control efforts [11]. RMT, previously referred to as “outdoor malaria transmission,” has been addressed by 2 specialized working groups over the years, the Roll Back Malaria Vector Control Working Group work stream on outdoor transmission (https://endmalaria.org/until-2015-%E2%80%93-outdoorresidual-malaria-transmission) and the Mekong Outdoor Malaria Transmission Network (https://www.apmen.org/events/7-10-november-2016), centered at Kasetsart University in Thailand. In this region, “outdoor transmission” is just one of several components encompassing RMT, owing to a combination of human and vector behaviors when forest workers, dwellers, or periodic, temporary forest exposed populations reside in or visit forest areas or do not sleep in protected dwellings or structures [9, 12–15].

In addition, local vector species may exhibit behaviors that allow them to avoid LLINs/IRS to which they are physiologically susceptible. According to Killeen [8], the primary vector behaviors that contribute to maintaining residual transmission are “(1) Natural or insecticide-induced avoidance of contact with treated surfaces within houses and early exit from them, thus minimizing exposure hazard of vectors which feed indoors upon humans; (2) Feeding on humans when they are active and unprotected outdoors, thereby attenuating personal protection and any consequent community-wide suppression of transmission; (3) Feeding upon animals, thus minimizing contact with insecticides targeted at humans or houses; [and] (4) Resting outdoors, away from insecticide-treated surfaces of nets, walls and roofs.” The current review focuses on the more obvious and readily surveyed issues where and when compromised exposure and unpreventable transmission risk may occur.

The purpose of this review is to synthesize the current body of evidence on vector and human behaviors that contribute to RMT and illustrate some of the complexities in using programmatic data and findings from research on vector control coverage, general health services, and methods for characterizing human-vector interaction. The review focuses on 2 selected ecoregions affected by persistent malaria transmission because of serious technical and health system problems [16], namely, (1) forest malaria in the GMS and in the Indo-Malaysian Archipelago, with variability in intensity of transmission, vector exophily, length of transmission season, and health system performance; and (2) coastal and lowlands malaria in the Oceania Region, with the potential (at least in principle) for mosquito habitat source reduction. 

Within each of these ecoregions, there is considerable variability related to anthropic, natural, and health system factors that affect the magnitude of RMT. It is also appropriate to contextualize the review around the revised RMT definition: “persistence of malaria transmission following the implementation in time and space of a widely effective malaria programme” [17]. This is derived from the 2014 WHO guidance note regarding transmission that occurs even with sufficient access to and usage of ITNs or well-implemented IRS, as well as in situations where ITN use or IRS are not practical interventions. 

Depending on local circumstances, these core interventions can be impractical when people are awake and active, with nets sometimes just a meter or two outside of their effective physical reach. LLINs and long-lasting insecticidal hammock nets may readily be used to protect forest workers sleeping outdoors but are generally impractical owing to the strenuous and demanding nature of forest work [12, 18] (Lucas Nene, personal communication, July 23, 2020) and represent an additional burden that not all forest workers are willing to accept [19]. Users associate remote settings with increased damage and/or soiling of bed nets, driving the choice of which type of bed net to bring (Lucas Nene, personal communication, July 23, 2020). Sleeping in makeshift lean-to or improvised ground-level shelters, rather than raised structured, often in hammocks, increases exposure of a high proportion of forest workers to malaria vectors [12, 20]. Hanging mosquito nets outdoors may be problematic given current commercial mosquito net designs and their reliance on external supporting structures. 

In Cambodia, some users hire local tailors to alter and sew multiple nets together into a larger coverage net (Lucas Nene, personal communication, July 23, 2020). User complaints include the difficulty in folding and storing the stiff/rough fabrics of LLINs and the fact that remote sleeping places are often constrained by obstacles and limited space; in remote settings, this promotes improvisation of hanging solutions, such as cutting poles and digging post holes to support free-standing bed nets (Lucas Nene, personal communication, July 23, 2020). Alternatively, in various sleeping areas or household structures, bed nets may be compact in size or hung using internal or complementary supporting structures or alternative “clamping/tying” systems, increasing their ease of use [21].

To address these concerns, the variables noted above for each ecotype must be defined. There is increased interest in malaria vector bionomic databases and human population movement, with information compiled from published literature [22, 23]. While the framework described by Guyant et al [24] has been used to develop more targeted behavior change and outreach interventions for mobile and migrant populations in Cambodia, a programmatic approach based on field observations is adopted herein. This approach is used to define 5 RMT categories: (1) migratory forest-goers of various types; (2) indigenous people who live in the forest, or forest dwellers; (3) village-based people who perform seasonal work in “farm huts”; (4) security, wildlife, and border protection and defense forces; and (5) people living in lowlands and coastal areas where *Anopheles punctulatus* complex species are present (Australasian Region).

While human *Plasmodium knowlesi* is primarily regarded a zoonotic pathogen, all indications suggest that human-to-human transmission can occur and probably is occurring in some situations [25]. Recent epidemiological trends in eastern Malaysia (Borneo) suggest that *P. knowlesi* infections will become more important as *P. falciparum* and *Plasmodium vivax* are eliminated [26, 27], and the close parallels with residual human malaria parasite transmission suggests the inclusion of “monkey malaria,” a term that is defined within a specific context of transmission epidemiology and involves several sylvatic vectors belonging to the Leucosphyrus group (*Anopheles dirus* and *Anopheles leucosphyrus* complexes—*Anopheles balabacensis, Anopheles cracens, A. dirus,* and *Anopheles latens* [28]). The human populations at greatest risk of infection are inhabitants of hilly forested areas, particularly ethnic minorities and subsistence farmers who have relatively substandard living conditions and low educational background, and whose normal life activities include forest exploitation and subsistence-level swidden cultivation practices [29–32].

## METHODS

### Search Strategy and Eligibility Criteria

This review follows the guidelines in the Preferred Reporting Items for Systematic Reviews and Meta-Analysis (PRISMA) statement [33]. The search occurred between January and May 2020. All data were extracted by 2 independent researchers, and discrepancies were resolved by consensus.

### Data Sources and Search Strategy

**Figure 1.**
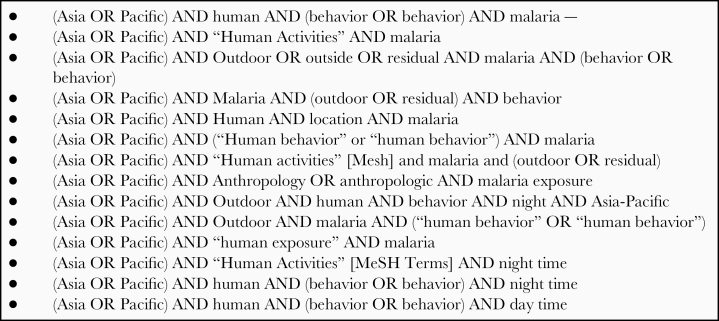
Key search terms used for systematic review.

Studies were identified by searching electronic databases, scanning reference lists of articles, and consulting with experts in the field. No limits were applied for language in case there was an available English translation. The search was applied to PubMed and the Cochrane Database of Systematic Reviews. The search terms in Figure 1 were applied to all databases.

### Inclusion Criteria

In accordance with the 2014 WHO (2014) definition of RMT—“All forms of malaria transmission that persist after full universal coverage with effective ITN and/or IRS interventions has been achieved” [9]—we used the following inclusion criteria to down-select abstracts and publications for the review: data on 1 ownership AND 1 usage indicator (relating to ITN/LLINs) OR data on the IRS indicator OR insecticide susceptibility of primary and secondary vectors OR human and vector behavior observations from qualitative and quantitative surveys, including nighttime human activities.

Indicators for ownership of ITN/LLINs included the percentage of HHs with at ≥1 ITN/LLIN and the percentage of population with access to an ITN/LLIN within the household. Indicators for usage of ITN/LLINs included the percentage of the population reporting having slept last night under an ITN/LLIN and the percentage of <5-year-olds with reports of having slept last night under an ITN/LLIN. The IRS indicator was the percentage of houses (population) protected by IRS in the last 12 months. Ownership of >1 ITN/LLIN per household and ITN/LLIN use in the target groups of children <5 years of age and pregnant women were key indicators for the Global Fund grants.

### Evaluation

Data were obtained from demographic and health surveys or malaria programmatic household surveys. One ITN per 2 people was considered to be sufficient, on average, to protect all individuals in the household [1, 2]. Where access to ITN/LLIN data is not available, we reviewed other indicators, such as (1) households with ≥1 net (any kind)/ITN/LLIN; (2) households with sufficient nets (any)/ITNs/LLINs; and (3) households that received IRS in previous 12 months; d) household with sufficient ITNs and/or IRS in previous 12 months.

According to Monroe et al [34], behavior is defined as “The observable response of a man or animal to a situation,” and the term is used broadly in this review to encompass human activities, location, and sleeping patterns. This includes activities occurring within or nearby the home, within the community, or outside of the community. We reported these activities from various publications that reported human behaviors in relation to malaria exposure. Specifically, studies included malaria-endemic settings in the 2 Asia-Pacific ecoregions and a description of human behaviors occurring when malaria transmission can occur, that is, when malaria transmitting vectors are active.

## RESULTS

### Search Results

**Figure 2.**
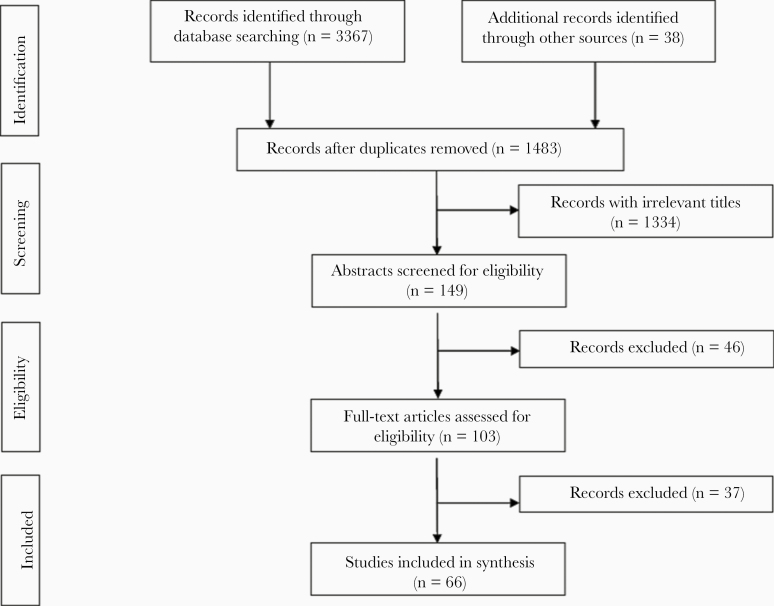
Flowchart showing sequence of database searches, identification, screening, and selection of included studies in the review.

The search results are illustrated in Figure 2. Initially, 3367 records were identified through database searches and 38 additional records were identified through other sources (eg, “gray” literature—unpublished reports, reference lists). After screening of title and abstracts, the remaining 103 papers were assessed and reviewed in full, after which 66 articles were included (Figure 2). The most common reason for exclusion in the final stage was the lack of a direct relation with or adherence to the RMT concept and an applicable result in malaria elimination settings.

### Study Characteristics

Selected studies were published between 1995 and 2020, proportionally representing Cambodia (15%), Papua New Guinea (14%), Vietnam (14%), Myanmar (12%), Malaysia (9%), China (9%), Solomon Islands (8%), Lao (6%), Thailand (6%), Indonesia (2%), and multiple Asia-Pacific countries (6%). The included studies fit into ≥1 of the 4 criteria: (1) ownership and usage of ITN/LLINs, (2) IRS application, (3) insecticide susceptibility of primary and secondary vectors, and (4) human and vector behavior observations focused on nighttime human activities. The selected articles emphasized the significance of epidemiological, entomological, sociodemobehavioral characteristics and drivers of RMT, and observations of insecticide susceptibility of *Anopheles* mosquitoes. Several review article on outdoor malaria transmission or RMT were used to complement the review [7, 31, 35–38].

Table 1 provides an overview of 14 studies conducted in the GMS and Asia-Pacific region. Given the study objectives, each example provides some recommended “policy” considerations together with challenges and opportunities. A key theme is the continuing burden and challenges that RMT poses in control areas, regardless if in an elimination phase or not. Mosquito and human behavioral patterns are major contributing factors for sustaining RMT. Across this wide geographic coverage is the inherent spatial and temporal heterogeneity in malaria epidemiology in which human and vector interactions occur. A better understanding of this complex relationship will be needed in order to develop and adopt better tools to combat outdoor transmission not affected by ITN use (or IRS) while promoting other bite prevention tools for those exposed.

**Table 1.** Selected Malaria Characteristics From Included Studies

Location and References	Population Type	Study Type	Objectives	Recommended Policy	Challenges and Opportunities
Cambodia: Gryseels et al, (2015) [39]; Durnez et al (2013) [40]; Bannister-Tyrell et al (2019) [41]	Forest-goers from 113 of the high-endemic villages in Ratanakiri Province (MalaResT Trial); people living in or near the forest fringe that use the forest for economic activities	Mixed method at 2 time points; sequential mixed-methods: quantitative survey research methods used to complement findings from qualitative ethnographic research; qualitative ethnographic	Ancillary work in larger trial to determine the effectiveness of mass use of topical repellents in addition to use of LLINs in controlling transmission as measured by community impact; understand how different populations, mobility, livelihood patterns, and activities within the forest intersect to potentiate malaria risk and affect the effectiveness of malaria control and elimination strategies	Reimagining malaria interventions by focusing not only on the heterogeneity in malaria transmission, but more specifically on the connection between varying human and vector behaviors, evaluate what works; what is still missing, and how to accelerate the progress in malaria control toward elimination; as forest groups often converge in the same areas, interventions targeting the vector population may have a potential role; ultimately, a multisectoral approach as well as innovative and flexible malaria control strategies are required for malaria elimination efforts to be successful	Transmission due to early and outdoor biting is among the major challenges; slash-and-burn farmers’ multiple residence system, locally used (partially) open housing structures, variance in labor and social activities, sleeping times, and bed net use; movement between different houses with varying levels of exposure to indoor and outdoor mosquito biting results in a constantly changing vulnerability to malaria; reported sleeping times vary according to the context; additional gaps in night protection, however, cannot be addressed with LLINs alone (eg, outdoor economic forest activities and toilet practices); most forest-goers had experienced multiple episodes of malaria and are well informed about malaria risk, but economic realities drive local residents to pursue forest-based livelihoods; severe constraints of available vector control methods mean forest-goers have limited capacity to prevent vector exposure; as they access the forest using many different entry and exit points, border screening and treatment interventions may not be feasible
Xieng-Ngeun and Nane districts, Luang Prabang Province, Lao PDR: Tangena et al (2017) [42]	Subsistence farmers and forest-goers	Rapid participatory rural appraisals and surveys with entomological surveillance	Assess the risk of exposure to vector mosquitoes in relation to different typologies of human behavior	Local people using DEET-based topical repellent, long clothing, and mosquito coils; medium risk of transmission in rubber plantations based on PSI work in Lao/Vietnam [43]	Visiting forests during the day has a higher risk of malaria vector exposure, but risk does not increase when working and living in the rubber plantations; need to broaden current vector control activities to include rubber plantations very near forests
Tha Song Yang District, Tak Province, Thailand: Edwards et al (2019) [44]	Forested foothills, subsistence farming, slash-and-burn agriculture beyond the villages	Cross-sectional behavior and net survey, observational and entomological collections in 2 villages and forested farm huts	Investigate the magnitude of RMT and contributing risk factors	Provide access to LLINs beyond village to farm huts and forest locations that are frequently visited by community members as these have a higher abundance of vectors and highest-risk practices	Novel personal protection tools that require minimal behavioral change and are accessible/affordable for the target populations (eg, = insecticide-treated blankets and clothing, spatial and topical repellents); use of entomological end points to show efficacy in low-transmission settings and acceptance of such tools in elimination strategies
Khanh Vinh District, Khanh Hoa Province, Vietnam: Edwards et al (2019) [45]	Subsistence farmers in rural villages and upland forest foothills practicing seasonal farming, slash-and-burn agriculture beyond the village	Entomological, epidemiological, and observational methods across 3 ecological sites frequented by village community, farm huts, and forest waypoints	Investigate how vector and human behaviors interact to contribute to RMT in an area poised for malaria elimination	Improve access to LLIHNs among forest-goers at risk of malaria and other vector-borne diseases through public-private partnerships	Evaluate new personal protection tools that will minimize behavior change, and highly accessible/feasible for use by population
Dabhine and Myothugyi areas, Rakhine State, Myanmar: Smithuis et al (2013) [46]	Cohort of 8175 children <10 y old from 22 villages	Cluster-randomized controlled trial to assess effectiveness of ITNs among 8175 children <10 y of age for 10 mo	Conduct entomological and population sleeping behavior surveys alongside multivillage ITN effectiveness study; observe incidence and prevalence of *Plasmodium falciparum* and *Plasmodium vivax* infections and the biting behavior of *Anopheles* vectors	Prioritize provision and access to early diagnosis and effective treatment; where such services are already in place and sufficient budgets available, including use of ITN can be a cost-effective integrated approach for control	Where malaria transmission is highly seasonal and unstable showing spatiotemporal variation, ITNs do not provide consistent protection against malaria in areas with weak secondary vectors; all major *Anopheles* vectors are characterized by early and outdoor biting, often before people are protected by an ITN
Malaysia: Kudat District, Sabah Barber et al (2013) [47]; Chua et al (2019) [48]; Grigg et al (2017) [30]	Secondary forest and small plantations of coconut, rubber, or oil palm; local swidden farming as primary occupation	Case-control, prospective clinical study to compare malaria risk factors, clinical spectrum, and outcome of severe disease by human and simian malaria, entomological and programmatic reports	Case-control study to assess human and environmental factors associated with zoonotic *Plasmodium knowlesi* malaria risk; entomological investigation to determine diversity and abundance of vector species in the 5 habitat types commonly found in rural areas of Sabah	Individual-level factors affecting zoonotic *P. knowlesi* transmission in established endemic areas are potential targets for future public health interventions, along with ongoing promotion of conventional malaria prevention activities	Novel tools are needed to address outdoor farming, vegetation clearing or plantation work, older males, sleeping outside, and travel history—for example, outdoor residual spray using a novel formulation of deltamethrin K-Othrine (PolyZone) (Rohani et al [2020] [49])
Papua New Guinea: Rodriguez-Rodriguez et al (2019) [50] and Rodriguez et al (2019) [51]	Coastal island atolls, lowland and highland populations from southern, Momase, islands, and highland regions	Mixed methods: cross-sectional malaria indicator survey (quantitative) and in-depth interviews and focus group discussions (qualitative)	Assess the role of human behavior in relation to malaria transmission and transmission heterogeneities by (1) identifying activities and livelihood relevant for malaria transmission, (2) understanding measures currently in use to prevent or reduce mosquito biting in the study sites, and (3) identifying behavioral differences between population groups	Study highlighted the potential of “outdoor biting” that can hamper malaria control and elimination efforts if not addressed appropriately because people spend a remarkable amount of time outdoors without protection from mosquito biting; complementary interventions to LLINs targeting groups, places and activities in order to prevent outdoor biting in the evening and understanding local community behavior are crucial to advance elimination	Need to integrate main study findings with concurrent entomological and malaria infection prevalence data to quantify behavioral risk factors of exposure and better quantify local transmission; given diverse transmission settings in PNG, the national control program must consider local heterogeneity when choosing interventions and ensure continuous monitoring of trends
East Sepik Province, Papua New Guinea: Kattenberg et al (2020) [52]	Wet tropical low hill forests, plains, riverine plains; rural villages (n = 2231 [2005] and n = 2348 [2013])	Cross-sectional community surveys, pre- and postimplementation observations using malaria prevalence; 2005: 73%; 2012–2013: 12.2%	Assess impact on malaria prevalence with control focused on mass distribution of LLINs	To further reduce transmission, additional surveillance approaches, novel tools and community engagement strategies may need to be combined with sustained LLIN coverage and effective malaria case management; understanding local heterogeneity and the key parameters (eg, LLIN coverage/use, socioeconomic factors, vector behavior, environmental factors) that drive transmission is essential to designing and implementing site-specific control strategies	Despite strengthening the MCP in Papua New Guinea and showing a substantial decrease in malaria prevalence, areas with high ongoing transmission remain; given local heterogeneity of transmission, identification and targeting focal points of persistent RMT are needed including development of sensitive and practical surveillance tools to identify and target high-malaria areas and households
Central Island Province, Solomon Islands: Pollard et al (2020) [53]	Coastal villagers—primarily subsistence agriculture and fishing	Experimental, observational, interviews and movement diaries	Study of people over a 14-d period and quantifying human-vector interactions where and when humans are exposed to the bites of vectors	IRS should include outdoor kitchens and verandas in addition to standard applications to inside walls; alternatively, novel control methods such as insecticide-treated durable wall linings, spatial repellents, insecticidal paints, and screening to mosquito-proof verandas and kitchens should be evaluated	Despite excellent access and near-universal use of indoor LLINs, a large protection gap exists with people exposed in the outdoor peridomestic area when many malaria mosquitoes are seeking blood meals

Abbreviations: DEET, N,N-diethyl-meta-toluamid; IRS, indoor residual spray; ITN, insecticide-treated bed net; LLIHNs, long-lasting insecticidal hammock nets; LLIN, long-lasting insecticidal net; MCP, Malaria Control Program; PDR, People’s Democratic Republic; PNG, Papua New Guinea; PSI, Population Services International; RMT, residual malaria transmission.

### Access to, Preference for, and Use of Vector Control Tools

Because access to ITNs is the primary driver of their use [54–57], we find variations in the reporting and sources of ITN access and use, and IRS coverage. This review did not include other indicators associated with performance of vector control interventions, for example, ITN/LLIN durability, IRS residual efficacy, Larval Source Management effectiveness, intervention coverage, preference, and populations having access to or use of health services. Several studies have found that individuals and household use untreated nets because of their perception that LLINs are made of coarse fabric, are too small, have easy-to-break mesh holes that are too large, wrinkle/shrink after washing, and have reduced efficacy over time [39, 58–60].

Among the 14 studies, 3 (in Cambodia, Papua New Guinea, Sabah in east Malaysia) use a combination of field research and programmatic surveys, while 11 (78.7%) relied on field research surveys. Eleven studies reported ITN ownership (numbers of nets distributed, persons per net, households with nets, or nets per person), and 3 studies (in Thailand, Vietnam, and Papua New Guinea) reported on population access to ITN in households (defined as the percentage of the de facto household population who could sleep under an ITN if each ITN in the household was used by up to 2 people) (see Table 2).

**Table 2.** Review of Determinants of Residual Malaria Transmission in Cambodia, Lao People’s Democratic Republic, Myanmar, Thailand, Vietnam, Papua New Guinea, and the Solomon Islands

Location and References	Ecotype, Annual Parasite Incidence (per 1000) or Malaria Prevalence, %	Vector Control			Human-Vector Interaction			
		Access to ITN or LLIHN, %	Use of ITN or LLIHN, %	IRS coverage, %	Human Behavior Methods and Information Collected	Entomological Methods	Timing of Entomology and Human Behavior Data Collection	Human Exposure to Malaria Vectors
Cambodia: Incardona et al (2007) [61]; Gryseels et al (2015) [19]; Durnez et al (2013 [40] and 2018 [62])	Forest plots and villages in Eastern region: Borkeo and O’Chum districts, Rattanakiri Province; ……………. Forest plots in Western region: Pailin province	LLIN ownership: 98.5% (Gryseels et al [19]); 68.4% (Rattanakiri); CMS for Rattanakiri^a^: 39.4 (2007), 63.7 (2010), and 99.3% (2013) …………… 69.2% (Pailin); 81.8% (Pursat) CMS for Pailin^a^: 38% (2007), 67.8% (2010) and 100% (2013)	70.7% (Forest workers) (Durnez et al [62]); 79.1% reported, 69.5% observed (Gryseels et al [19]); 95% (Durnez et al [62]) …………. 66.3% (Forest workers)	ND	In-depth interviews and participant observations.in Ratanakiri Province; CMS: households interviews using pretested questionnaires	Outdoor HLC in 2 intervention and 2 control villages; from 1700 to 2200 h, 1700 to 0800 h, and 1900 to 0600 h	Rattanakiri—survey 1 (4 surveys): July–August 2009, July–November 2010, July–August 2011; survey 2 (8 surveys) every 2 mo between April and October 2012 and 2013	Early human-biting proportion of *Anopheles dirus* s.l. in villages: 39%–48% and forest: 24%–26% (Pursat); *Anopheles minimus* s.l./*Anopheles aconitus* and *Anopheles maculatus* s.l.: 54.4% and 56.6% (in Rattanakiri); 26.9% and 32.9% (villages in Pailin and west Pursa), respectively; EIR before 2200 h: 0.52
Xieng-Ngeun and Nane districts, Luang Prabang Province, Lao PDR: Tangena (2017) [42] ……………... Laman District, Sekong Province, Lao PDR: Vythilingam et al (2003 [63] and 2005 [64]); Nonaka et al (2010) [65]; Killeen (2014) [8]	4 Rural habitats: secondary forests, mature rubber plantations, immature rubber plantations, and villages; API: NA, as few malaria cases were imported every year; forest clearing in river valley landscape, fertile plain patterned with a patchwork rice fields and mixed fruit orchards; parasite prevalence: 10.5%–11.8% (2000)	90% (Ownership) ……………... August 2008: 2 ITNs per household	NA ……………… August 2008: 94%	ND ……… ND	Rapid participatory rural appraisals to study daily and monthly activities of the rubber workers and villagers ……………….. Cross-sectional surveys, questionnaire-based interviews, blood examinations among farmers and household members	Outdoor human double-net trap (HDN) during day (0600–1800 h) and evening (1800–0600 h) …………………... Indoor and outdoor HLC, 1800–0600 h	Mosquito collections: 9 mo, from July 2013 to July 2014; human behavior studies: November 2013 and July 2015 …………………….. HLC in August and October 2000, April and October 2001; household surveys March (dry season) and August (rainy season) in 2008	Exposure to malaria vectors is 1.3 times (95% CI, 1.2–1.4) higher in forest habitats; lower risk of malaria in rubber plantations at night (OR, 0.9; 95% CI, 0.8–1.0), living and working in rubber plantations (0.6, 0.4–1.0), or staying in villages …………………………………... Staying overnight in farming huts was not associated with an increased risk of malaria infection in settings with ITNs widely used in farming huts (Nonaka et al [65]); proportion of human exposure to *A. dirus* that occurs indoors for both unprotected residents (πh,i, 0.91) and LLIN users (πh,i,n, 0.4) (Killeen et al [8])
Tha Song Yang District, Tak Province, Thailand: Edwards et al (2019) [44]	2 Villages, 1 hamlet; API: 148 and 278; parasite prevalence: 0.27%–0.89%	Population access to an ITN: 80.5%	Adults: 79.5%; children aged 5–18 y old: 82.5%	71.4%	Transect surveys; direct observations and qualitative analysis using GPS trackers	Indoor and outdoor HLC and cow-baited trap 1800–0600 h	Mosquito collections: June–November 2016; Malaria survey: September 2016	Indoor exposure 88%–93% for LLIN nonuser and 33%–45% for user Indoor users of LLINs during median sleep time: 45%–67%
Son Thai, Khanh Hoa Province, Vietnam: Edwards et al (2019) [45] …………….. Nam Tra My district, Quang Nam Province, Vietnam: Thanh et al (2015) [66] …………….. B Giap Map national forest, Binh Phuoc Province, Vietnam: Son et al (2017) [67]; Ngo et al (2014) [68]	1 Village, farm huts, forest plots; malaria prevalence: 1.71% (unpublished data, NIMPE) ……………… 4 Villages in a remote forested valley; prevalence: 7.8% (range, 3.9%–10.9%) ……………… Conservation park of rare and precious fauna and flora species, 260 km^2^, 700 m asl; Pf incidence of ranger population: 479/1000/y	Population access to ITN in HH: 91.5% ……………... % households with nets^b^: 23.3%; % HH with 1–2 persons per net: 6% …………….. NA	Farm huts: 44.4%; forest: 12.1%; regular use in farm huts: 72.7%; forest: 25% ……………… ND ……………… All rangers tend to sleep in hammocks with or without bed net	2.9% ……… Regular IRS due to unpopularity of bed nets ……… NA	Transect surveys; direct observations and in-depth interviews; GPS trackers; record time sleep and wake-up time ……………… Malaria survey and interviews on the different outdoor activities in and outside the community, sleeping habits, and malaria prevention measures ……………….. ND; feasibility study of malaria prophylaxis	Indoor and outdoor HLC, 1800– 0600 h; after July 2016: 1600–0600 h; cow-baited net trap: 1800–0600 h …………………... ND ………………….. Indoor CDC light traps; outdoor and indoor HLC from 1800 to 0600 h	Mosquito collections: June–November 2016 survey; September 2016 ……………………... No entomological surveys …………………… Parasitological and clinical surveys; mosquito collections: May–September 2016	Outdoor biting in the forest and indoor biting at the farm hut were highest during 20:00–21:00 h; 48% of biting by mosquitoes occurred before 9 PM in the farm huts: 45% of A. dirus (s.l.) and 100% of A. maculatus (s.l.) biting) …………………………………... ND ………………………………… *A. dirus* (84%) was most prevalent and preferentially anthropophilic, biting outdoors before 2200 h (similar to Trung et al [2005] [69]); exposure not estimated
Ma Noi and Phuoc Binh communes, Ninh Thuan Province, Vietnam: Erhart et al (2005) [70]; Van Bortel et al (2010) [71]; Thanh et al (2019) [66]; Grietens et al (2010 [72] and 2012 [73]) …………….. Phuoc Chien Commune, Vietnam: Sanh et al (2008) [74]	Ra-Glai villagers along the road with a second home at their slash-and-burn fields in the forest Parasite prevalence: 13.3% ……………… Phuoc Chien commune: malaria positivity rate: 32.1% (2003) and 15.5% (2006)	Median coverage: 2.5 people per bed net; December 2004: 7000 LLIHNs individually distributed to all residents (≥10 y old) in intervention clusters for 70% coverage of intervention population ………….. Households with ≥1ITN: central region, 18.9%; south, 8.9% (DHS [83])	Bed net use in village: 84.6%; in forest fields: 52.9%; overall LLIHN use at night in village: 56%; in forest fields: 20.7% (evening) and 6.4% (at night) ……………… Children <5 y old who slept under an ITN the previous night: 25.1% (central) and 7.4% (south) (DHS [83])	NA ……… Remote garden plots were not sprayed	Mixed-methods study integrating qualitative data from focused ethnography and quantitative data during malariometric cross-sectional survey ……………… ND entomological and mass blood surveys	Indoor and outdoor HLC, 1800–0600 h: locations of collections: in villages (2 houses), forest (2 sites), and “on the way” (in between) …………………... Phuoc Chien commune: outdoor HLC and indoor CDC light traps from 1800 to 0600 h	1st survey: November 2004; 2nd survey: October and November 2005; 3rd survey: October–November 2006 …………………….. Parasitological and entomological surveys in Phuoc commune: September–October 2006	Highest biting activity of *Anopheles* vectors occur in the evening, with 6% of bites by 1900 h, 25% by 2000 h, and 50% before 2200 h; correlating human/mosquito activity patterns with the proportion of people protected by either LLIHNs and/or ITNs, local farmers at the forest fields are exposed to mosquito bites mainly owing to low ITN use; while half (52%) of Ra-glai respondents were asleep by 1900 h, only 58% would regularly be protected by ITNs; among the fraction of people staying out later in forest fields only about 20% were using LLIHNs; comparatively, at villages, both people staying out late and those sleeping were more likely to be protected, respectively by LLIHNs (56%) and ITNs (92%); Phuoc Chien: probable malaria transmission in the garden plots due to presence of *A. dirus*, a species absent in the village 2 km away
Dabhine and Myothugyi areas, Rakhine State, Myanmar: Smithuis et al (2013) [46, 75]	Coastal plain area (without hills or forest, where rice and other crops are cultivated); rice fields and partly forested hills	Complete coverage; approx. 1.6 ITN per child (5000 ITNs distributed in April 1998)	84% in ITN group; 7% slept in untreated nets in control group	ND	Interview all ITN recipients, during cross-sectional surveys on ITN usage and ITN washing habits	Indoor and outdoor HLC (“HBC”), 1800–0600 h and 1700– 0700 h (3rd survey); cow-baited net trap (night and morning); exit traps; indoor knockdown collections	1st survey: November 1995 to April 1996; 2nd survey: July 1996 to April 1999; 3rd survey: December 1999 to January 2000	Biting-risk of 0–4- y-old children in no-net villages was about 61% and 91% compared with average (all age groups combined); compared with an average person in a village without ITN, the risk of mosquito bites per infant was 19% and 89%, respectively; proportion of human exposure to mosquitoes indoors for unprotected residents (πh,i): 0.41 (A.* epiroticus*), 0.48 (A.* subpictus*), and 0.54 (A.* annularis*); proportion of human exposure to mosquito that occurs indoors for LLIN users (πh,i,n): 0.06 (*A. epiroticus*), 0.06 (*A. subpictus*), 0.07 (*A. annularis*) (Killeen et al [2014] [8])
Sabah State, Kudat District, Malaysia: Grigg et al (2017) [30]; Chua et al (2019) [48]; William et al (2013 [26] and 2014 [27]); Manin et al (2016) [76]	Paradason, Kudat District, Sabah, East Malaysia API (2011): 14.3 per 1000 ecological; habitats: forest edge, playground: long house, oil palm plantation, and bush shrubs (Chua et al [48])	Study sites in Timbang Dayang, Limbuak Laut (Banggi), and Paradason (Kudat): 1.65 people per ITN; population access to ITNs in HH: 121%	NA	100% (128 Houses in 2013; 144 houses in 2014)	Questionnaires and household surveys for case-control study using demographic, social, behavioral, household, and environmental variables associated with malaria risk	Outdoor HLC: 1800–0600 h	Mosquito collections: 14 mo. from October 2013 to December 2014	Sleeping outside was an independent acquisition risk factor (aOR, 3.61; 95% CI, 1.48–8·85; *P* = .005), as was a history of recent travel (2.48; 1.45–4.23; *P* = .001); sleeping outside in the forest or plantation during a trip was not significant; use of a bed net remained unassociated with protection; a history of activities in the forest were not significantly associated with increased *Plasmodium knowlesi* malaria risk, or any other specific recreational activities such as hunting; outdoor residual spraying of houses reduced the simian vector population and simian malaria transmission (Rohani et al [2020] [49])
Papua New Guinea: Rodriguez‑Rodriguez et al (2019) [50]; Kattenberg et al (2020) [52]; Hetzel et al (2016) [77]; Reimer et al (2016 [78] and 2013 [79])	Coastal villages with coconut plantations, swamps inland foothills with thick vegetation; incidence: 20–115 per 1000 (in 2010) to 1–47 per 1000 (in 2014)	Momase: 39.4 (2008–2009), 75.4 (2010–2011), and 79.0 (2016–2017); Islands: 44.0(2008–2009), 98.3 (2010–2011), 77.1 (2016–2017); access to an LLIN (2016); Madang: 79.8; East Sepik Province: 71.9%; New Ireland: 78.7%	Momase: 47.0 (2008–2009), 48.4 (2010–2011), 70.2 (2016–2017); Islands: 25.4 (2008–2009), 40.0 (2010–2011), 38.5 (2016–2017); Sausi: 90%–100%; Dreikikir: 77%–86% and 79% (3-mo interval); Madang: 54% and 79% (3-y interval) (Reimer et al [78])	Nil	Hourly observations on number of animals and additional people present in hamlet (Reimer et al [78])	Outdoor HLC 1700–0600 h	Mosquito collections for 1 y before and 1 y after nationwide LLIN distribution; Madang: August 2008 to November 2011; Dreikikir: September 2008 to July 2011	Significant decrease in human landing rates in the year after LLIN distribution and remained low through y 3; LLINs may still have a large communal impact if LLIN coverage and usage is high, including individual use and high community LLIN coverage (Hetzel et al [2015] [86]; Reimer et al [78]) Aging of nets, early and outdoor mosquito biting may fuel ongoing transmission [Rodriguez-Rodriguez et al [50]
Solomon Islands: Pollard et al (2020) [53]; Russell et al (2016) [80–82] DHS (2015) [83]	Coastal islands; API: 280	≈79.3% approximately	84%	≈26.2%	Daily movement diaries, interviews, and direct observations	Indoor and outdoor HLC, 1800–0600 h	Mosquito collections: July 2012	Almost universal access to and use of LLIN with, only 7% of people were under an LLIN during the 1800–2100-h peak biting period when 76% of *Anopheles farauti* bites occur; proportion of exposure to mosquito bites on humans occurring indoors (πi): 0.130 ± 0.129.
Thailand: Somboon et al (1995) [84] and 1998 [85])	Forest and forest fringe areas in Mae Sarianp District; Mae Hong Son Province; API (1989): 80.7–279.7 per 1000 (1989)	ITN coverage: >80%; 2.2–2.7 person s per net	>90%	IRS suspended from October 1989 until the end of the study	Fortnightly household interviews and observations of human behavior during transmission season	Indoor and outdoor HLC, 1800–0600 h; CDC light traps, cow- or buffalo-baited net trap	Mosquito collections: monthly May–December 1990 and May–December 1991	Residential villages, farm huts and forests are sites of transmission; malaria risk for forest activities is 4–6 times higher than other activities and 13 times higher than staying in villages; higher biting density of vectors at the farm huts but similar inoculation rates between villages and farm huts; community-wide use of ITNs did not generally reduce the vectorial capacity of vectors in this area, probably because of the biting behavior of the mosquitoes

Abbreviations: aOR, adjusted OR; API, Annual parasite incidence; ASL, above sea level; CDC, Centers for Disease Control and Prevention; CI, confidence interval; CMS, Cambodia malaria survey; DHS, Demographic and Health Survey; EIR, entomological inoculation rate; GPS, Global Positioning System; HBC, human biting collections; HDN, Human double net; HH, Household; HLC: human landing collection; IRS, indoor residual spray; ITN, insecticide-treated bed net; LLIHN, long-lasting insecticidal hammock net; LLIN, long-lasting insecticidal net; NA, Not available; ND, not done; NIMPE, Vietnam National Institute of Malariology, Parasitology and Entomology; OR, odds ratio; PDR, People’s Democratic Republic; RMT, residual malaria transmission; PF, Plasmodium falicparium; s.l., sensu lato.

^a^Ownership is defined as having possession of ≥1 ITN

^b^The ownership of nets was reported without indication of age, condition or whether they were insecticide treated or not

While the Cambodia Malaria Surveys (Table 2) in 2004, 2007, 2010, and 2013 provided national results for ownership, access, use, and the use-access ratio, they conceal variations by region, which may result from differences in survey timing vis-à-vis the rainy season (among other reasons) From Table 2, it can be seen that ITN access (calculated as having ≥1 ITN in the house) has incrementally approached universal coverage target between 2007 to 2013 Overall, Cambodia had an excellent use-access ratio (0.95) in 2005, with higher ratios in Mondulkiri and Rattanakiri (1.11), Pursat (1.28), and Battambang and Pailin (0.97).

While Cambodia’s 2005 Demographic and Health Survey (DHS) contains information on malaria and ITNs, its 2010 and 2014 DHS surveys do not. Cambodia implemented mass ITN distribution in 2012 [13]. These outcomes were probably attributed to repeated free ITN distribution campaigns in Cambodia and Papua New Guinea [86] and across Asia-Pacific [36], which led to significant increases in mosquito net ownership and use-access ratios with few exceptions (Table 2), in combination with increased surveillance and testing, treating, and tracking over the last 5 years. In addition, strong commitment from policy makers and effective partnerships have created a catalytic effect on program success, resulting in 75% and 93% drops, respectively, in reported malaria cases and malaria deaths in GMS countries between 2012 and 2017 [87]. In view of case increase (resurgence and outbreaks) due to drug stock-outs, low use of mosquito nets, disruption to the village malaria workers program, and high influx of people into forest areas, where access to treatment is limited, a combination of approaches is needed to engage these hard-to-reach populations [38, 88, 89].

Before ITN rollout and mass distribution campaigns during the early 2000s in GMS countries, malaria transmission hot spots occurred within the village in southeastern Thailand, in groves of rubber and fruit trees where *A. dirus* sensu lato (s.l.) is the primary vector [90], and villages near the Thai-Myanmar border in Tak province [91, 92]. In the southeastern region [93], it was reported that transmission probably occurred in forest and rubber plantations rather than rice fields. In the latter region (Mae Hong Son district), entomological and epidemiological data suggest that residential villages, farm hut settings, and forests were transmission sites, but the status of forest foci was inconclusive [94]. 

For a long time, the consensus has been that mature rubber plantations, especially those next to the forest, provided a suitable habitat for *A. dirus* [95]. Results of unpublished parasite prevalence surveys conducted sporadically over the last 20 years in Cambodia [96–99], however, suggest that there is very little transmission in most plantations. Informal field observation by malaria veterans from Thailand and Vietnam indicates that the vast majority of cases among plantation workers are contracted in nearby forests where the workers go to collect forest products to supplement their income (see below). There has actually been an increase in the number of cases among some rubber plantation workers in recent years, but this has coincided with a global reduction in rubber prices, which has affected wages, forcing workers to find additional sources of income—forest products (Sean Hewitt, unpublished data). During the last 10 years, large-scale distribution and rollout of ITNs have provided effective community protection in residential villages that are accessible by central or district teams; however, the current tools are not sufficient because of human or mosquito behavior, and thus malaria transmission in Thailand, Papua New Guinea, and Solomon Islands is not residual according to the current definition, or is probably misclassified.

In situations where malaria transmission can be considered residual, because of the very good coverage of ITNs, as in Vietnam and Cambodia, persistent malaria transmission has moved from villages to farm plots and forests, with secondary vectors, such as *A. hyrcanus, A. barbirostris* sensu stricto (s.s.), *A. barbirostris* clade III*, A. nivipes*, and *A. peditaeniatus* exclusively captured in cow-baited traps [100] or with both primary and secondary vectors [44]. In such places, the deployment of ITNs needs new approaches, but other vector control tools are also needed.

Data on subgroups such as wealth quintile or urban/rural residence, which were not available for this review, may offer ways to identify target groups that do not use their available nets to the fullest degree. Nine of 14 studies (64.2%) reported ITN use, and 6 (42.8%) reported IRS coverage. For example, the 2013–2014 ITN distribution report from Kudat District, Sabah (target of “universal coverage” if the household owned ≥1 net for every 2 people) showed that 325 nets were distributed to 537 household members in 3 villages (Paradason, Limbuak Laut, and Timbang Dayang), giving an ITN access of 121% (650 of 537) which is “too many” because the household owned ≥1 net for every household member (H. Tanrang, personal communication, May 21, 2020). Interestingly, mosquito bed net use did not seem to be protective against *P. knowlesi* acquisition [30], consistent with findings from the same study area describing earlier peak biting times of *A. balabacensis* in the early evening (from 1800 to 2000 hours) as mosquitoes adapt to human bed net use later in the night, with biting mainly occurring outdoors [101]. However, given that lower levels of mosquito biting have been reported to continue throughout the night [101], the potential of indoor transmission remains. Consequently, the use of conventional prevention activities supported by social behavior change and communication efforts remains relevant, especially for the large proportion of people who did not use a bed net during travel away from home.

Three countries (Thailand, Vietnam, and Papua New Guinea) reported an ITN/LLIN use-access ratio, which provides an estimate of the proportion of the population using nets, among those who have access to a net within their household. This indicator clarifies whether a gap in net use is related to behavior or to lack of access to nets.

Given the apparent data deficiencies, it is important for National Malaria Control Programs (NMCPs) to monitor intra-household access to ITNs/LLINs and indicate whether gaps in protection are due to a lack of access to nets or to human behavior (Peeters Grietens et al 2019, [102]) Free access to LLINs is the primary determinant of participation in bed net distribution campaigns. Preference for larger nets and darker-colored polyester nets over polyethylene nets, owing to wrinkling and shrinking of the latter, has been noted in Solomon Islands [103], while a similar study in Vanuatu found preferences for larger mesh and wider nets [104]. Participants in Timor-Leste did not have enough experience with nets to make preferences revealing, because different brands of LLINs have the same color [14]. The shape of nets can influence their use. In Sri Lanka, the odds of LLIN use were 5.6 times higher for conical nets compared with rectangular nets among 530 LLIN-owning households. The preference vote was evenly split between brands (Yorkool and Olyset), with 505 conical and 155 rectangular nets selected. Respondents cited heat or lack of mosquitoes as the main reason for not using a net [105].

A recent qualitative study among end users in Cambodia showed clear partiality for softer fabrics (in this case polyester over polyethylene) [60]. Because users have access to a variety of bed net options, either from local markets or via free distribution programs, they have come to understand that mass distribution allows them to create whatever value they wish from the free nets. Thus, various alternative uses for distributed net can result, even far from their intended purpose of preventing mosquito-borne pathogens (eg, fishing, protecting fruit and vegetable crops from animal damage). Despite widespread distribution both from local markets to community-wide distribution programs, the product attributes of LLINs confine them to a narrow spectrum of preferred use for prevention of mosquito bites: (1) single-occupant users, often men; (2) use in remote settings, such as forest, mountains, and plantations. where chemical protection is highly valued against a perceived increase in mosquito threats; and (3) use in settings where versatility and durability is important. Whether polyethylene or polyester fabric, nets frequently targeted for household use generally lack the favorable product attributes necessary to provide a more comfortable experience users seek, as recorded in the in Solomon Islands [106], Cambodia [39, 58], Vanuatu [104], and Timor-Leste [14]. When these product attributes are coupled with a lack of user education and distribution strategies leading to oversupply, users find new ways of assigning value to the LLINs—often leading to premature abandonment or intentional misuse (Lucas Nene, personal communication, July 23, 2020).

Programs that wish to explore the relationship between various net attributes, preferences, and use rates should include questions from a recent literature review [107] and the “Malaria Matchbox” tool [108]. Survey questions can be complemented with well-designed, site-specific qualitative research for assessing existing products and services under consideration, current usage behavior, and the cultural context behind these behaviors. Irrespective of sample sizes, these more detailed studies can help reveal blind spots for improving future survey designs (Lucas Nene, personal communication, Jul 23, 2020). Such qualitative data assisted Cambodia to receive an exceptional waiver from GFATM to purchase the most preferred products by the community, with future quantitative studies on usage and acceptance to be based on net characteristics that have been suggested to enhance the usage among high-risk groups [59].

The relatively low IRS coverage (42.8%) in our review was not far removed from that reported in the Asia-Pacific region (proportion of structures or household sprayed, 67%; proportion of the population at risk protected by IRS, 56%) [36]. Given the relevance of this indicator in foci investigations, it is recommended that the NMCP should monitor IRS performance to ensure spraying top-ups/mop-ups during each spray round.

### Human and Vector Interaction

Human behavior is a central component of RMT owing to the overlap with vector behavior to allow better targeting of vector control to the human-vector contact point, especially where transmission occurs away from home (eg, the farm hut, forest, and forest fringe [39, 44, 45, 70, 71, 109, 110]). Among the 14 studies, 5 (in Cambodia, Lao PDR, Thailand, and Vietnam) used a combination of entomology and mixed methods (eg, in-depth ethnography, rapid participatory assessment, qualitative and quantitative, observations, and transect walks); 4 (in Lao PDR, Thailand, Myanmar, and Vietnam) used cross-sectional household interviews and entomology; 1 (in Solomon Islands) used movement diaries, observations, and entomology; 1 (in Papua New Guinea) used observations, household interviews, census of humans and animals, and entomology; 1 (in Sabah and Malaysia) used household surveys and pretested questionnaires in a case-control study of *P. knowlesi*; and 2 (in Vietnam) used entomology (see Table 2). Twelve studies (92.8%) conducted entomological surveys using all-night outdoor and/or indoor human landing collections, animal-baited and exit traps knockdown collections, or light traps; only 1 study (Lao PDR) conducted 24-hour mosquito collections using a human double-net trap.

 Ten studies integrated human behavioral and entomological data to provide a quantitative estimate of human-vector interaction occurring indoors and outdoors (Table 2). Of these, 4 studies used early human-biting proportion calculated as the percentage of vectors biting before 2200 hours (or the locally appropriate sleeping time) by vector species (1 for Cambodia and 3 for Vietnam) and the entomological inoculation rate for the respective parasite (*P. falciparum* or *P. vivax*) (2 in Vietnam). Four studies(3 in GMS and 1 in Solomon Islands) integrated human and vector data estimates of indoor and outdoor vector biting as well as the distribution of people indoors and outdoors for each hour of the night to produce a weighted estimate of exposure occurring indoors and outdoors. With the exception of Lao PDR, which used assumptions of community sleeping time and outdoor activity, this analytical approach was successfully used to quantify human-vector interaction by Killeen et al in rural Tanzania [111] for Myanmar, Thailand, and Solomon islands. Interestingly, the unusual late nocturnal, indoor-feeding behavior of *A. dirus* s.l. [8] is due to premass distribution of ITN/LLIN, which in the prolonged presence of impregnated nets, contributed to 24.4% and 26% of earlier outdoor biting in forested villages of Lao PDR (Vythilingam et al 2005, [112]) and Cambodia (Durnez et al 2013, [40]), respectively before sleeping time. Finally, 2 of the 10 studies documented odds of exposure from sleeping outdoors in the forest or plantation (Sabah) and habitat types (Lao PDR).

Two studies (in Thailand and Vietnam) used entomology, observations, and/or interviews to determine location-specific hot spots. One study reported the effects of pre-ITN and post-ITN distribution on human biting rates (Papua New Guinea), and 1 entomological study (Vietnam) did not quantify human exposure to vectors.

Although IRS increased the outdoor biting rate of *A. dirus* s.l. [113, 114], and *Anopheles minimus* s.l. in forested and foothill regions in Thailand [113, 115], *A. dirus* displayed avoidance behavior by not entering sprayed houses and resting on sprayed surfaces [114]. Twice as many *A. dirus* exited via window traps after spraying compared with DDT prespray structures [113], owing to the pronounced excitatory-repellent effects of DDT, and resulted in an overall greater reduction in human landing activity [116]. If mosquitoes are killed quickly, they may not have the time or ability to exit, so high mortality rates may result in apparent reduced exiting time [117]. DDT reduced the likelihood of attempted mosquito blood feeding by more than half compared with mosquitoes in the presence of deltamethrin, by causing a greater increase in the rate of mosquitoes exiting experimental huts or local houses (ie, owing to excitorepellency action) [116]. However, this behavioral avoidance action did not translate into an equivalent elevated risk in malaria transmission for *A. minimus* s.l. and *A. dirus*, both outdoor biting species. [113, 118], in forest fringe and rubber plantations, respectively. 

In the absence of knockdown or mortality data of mosquitoes entering experimental huts or houses, it is speculated that a higher survival of exiting mosquitoes that evaded contact with treated surfaces may sustain persistent transmission outdoors. Very few studies (in Thailand, Vietnam, Lao PDR, and Solomon Islands) recorded human behavior during human landing collection of the number of people present or actively using interventions at the collection site (either outdoors or indoors). In forested areas in the GMS and coastal islands in the southwest Pacific, measurements were made during the same periods and locations as vector biting with concurrent observations on hourly human activity, movement and use of ITN. Analysis of ITN use every hour and/or IRS status coupled with vector behavior observations and/or insecticide resistance status are useful to identify gaps in protection [119].

These 4 studies provide adjusted human biting rate (calculated as the product of human biting rate by proportion of humans observed inside vs outside, awake vs asleep, with vs without a ITN), which is useful to analyze human behavior together with vector behavior and use of vector control interventions [119]. For example, for an unprotected individual, comparing the proportion of vector bites occurring indoors with proportion of bites occurring outside provides an idea of relative exposure risk and is useful for characterizing residual transmission in a programmatic context.

**Figure 3.**
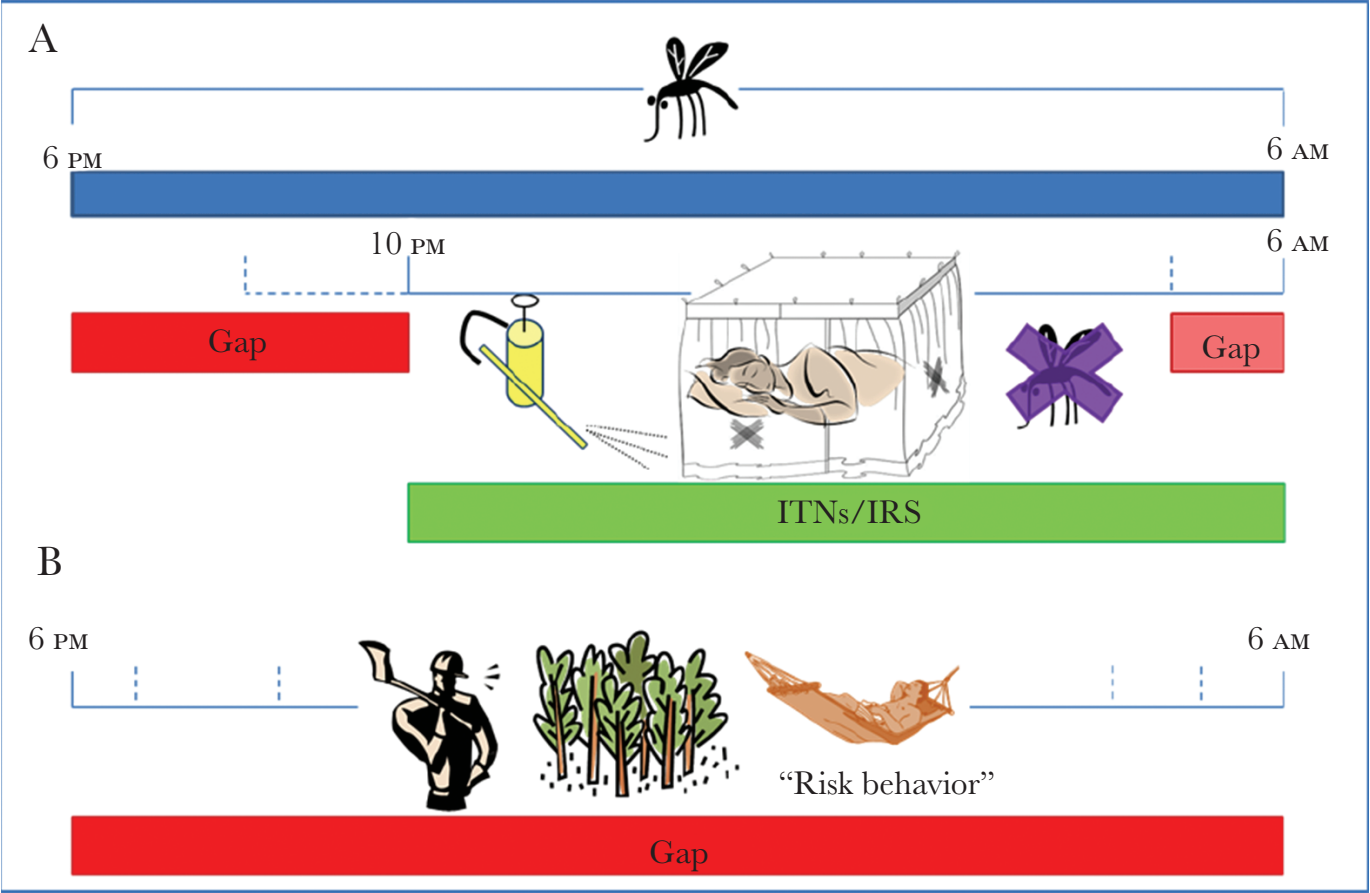
Protection “gap” when only indoor insecticide-based vector control measures are applied (adapted from Durnez and Coosemans [7]). For anophelines that blood feed both indoors and outdoors, the overwhelming majority of exposure events for an unprotected person may still occur indoors if mosquitoes actively seek blood throughout the night when most people are asleep inside their dwellings (*A*) or conducting outdoor activities during the night or early morning hours (*B*). Critical, site-specific data for contemporaneous entomological and human behavioral elements for quantifying the distribution of human exposure to malaria vectors across times of the night and indoor versus outdoor locations include entomological and human data. Entomological data at the local level include (1) directly comparable measurements of hourly indoor and outdoor biting rates by individual vector species over the full period of feeding activity and (2) reference estimates for the personal protection provided by insecticide-treated bed nets (ITNs) while they are actually being used, expressed in terms of proportional human blood feeding reduction. Human data at the local level include (1) local estimates of the proportions of the population who are indoors versus outdoors for each hour of the night (1800 to 0600 hours), (2) estimates of the proportion of population who are retired (asleep or trying to sleep) versus awake and active, for each hour of the night; and (3) estimates of the proportion of population using an ITN for each hour of the night [120]. Abbreviation: IRS, indoor residual spraying.

Similarly, very few studies (see above) recorded human behaviors and activities by location, resulting in the lack of “gap” in protection, not only before sleeping time, but also for people who remain outdoors during the night (Figure 3). Quantifying and characterizing gaps in personal protection against mosquitoes, defined as the proportional reduction in biting exposure an individual experiences as a direct result of personal using a protection measure, requires information on the behaviors of vectors and humans, as well as when and where they intersect. Vector-human interaction is useful for determining how this gap needs to be tackled by additional vector control measures and an integrated perspective on relevant indicators of human-vector interactions.

By applying Global Positioning System trackers (Thailand, Vietnam) or movement diaries (Solomon Islands) across time and relevant geographic areas to track population movement (eg, sleeping in villages vs sleeping at farms), it is possible to specify the exact location of transmission hot spots. Reports from Institute of Pasteur Cambodia of 20% daytime biting by Anopheles females during 24-hour static landing catches have implications for the protection of workers and among those moving from place to place, foraging for forest products between dawn and dusk. Similarly, reports from Lao PDR suggest that a recent outbreak in Nong District in Savannakhet Province, which affected males and females from all age groups, was the result of entire families foraging in the forest during the daytime for scarce but high value medicinal leaves to sell to Vietnamese buyers (Sean Hewitt and Bouasy Hongvanthong, unpublished information).

In summary, the deficiencies identified in this review that will improve the characterization of RMT are (1) sleeping or awake time (proportion of individuals asleep vs awake, indoors vs outdoors by hour during biting times) and (2) adjusted human biting rate (calculated as human biting rate times the proportion of humans observed inside vs outside, awake vs asleep, with or without a ITN/LLIN). Given that approximately 20% of *Anopheles* vectors were caught during the daytime in forested areas of Cambodia (Amelie Vantaux, personal communication, April 20, 2020), consideration should be given to daytime biting by opportunistic vectors, especially by moving landing catches that aim to replicate foraging behavior [15].

### Nighttime Activities, Personal Protection, and Transmission Risk Factors

Among 10 studies that covered some description of daytime and nighttime activities occurring during times when local malaria vectors are active, 6 (from Cambodia, Lao PDR, Thailand, Vietnam [2 studies], Papua New Guinea, and Solomon Islands [2 studies]) gave varying detail regarding locations of people and/or activities taking place in the peridomestic setting (inside and directly outside the home), as well as away from home, throughout the night. This included routine household chores and entertainment occurring in the evening hours before bed, routine livelihood activities that lasted throughout the night or part of the night, such as hunting, forest work, gathering forest products, logging, rubber tapping, supervision of local agroindustry, security, and sociocultural events (eg, funerals that lasted throughout the night) (Table 3).

**Table 3.** Review of Nighttime Human Activities, Categories, and Use of Prevention Measures (Adapted from Monroe et al [34])

Location and References	Methods Used to Record Nighttime Activities	Nighttime Activities Identified and Prevention Methods Adopted	Nighttime Activity Categories
Cambodia, Rattanakiri Province: Gryseels et al (2015) [19]; Durnez et al (2018) [62] Stung Treng: Sanann et al (2019) [121]	A first visit, in the evenings between 1900 and 2100 h depending on the availability of the household, consisted of the observation of housing structures, people’s resting behavior, bed net characteristics, and topical repellent use of all household members; as actual bed net use at night might not be directly observed, bed nets that were suspended in the evenings before bedtime with ≥2 corners were considered ready for use	Basket weaving, collecting water and firewood, tending to cattle, watching television, being bitten by mosquitoes when urinating and defecating at night or in the early morning (Gryseels et al [19]); hunting, forest work between 0400 and 0800 h and between 1900 and 2300 h, socializing with alcohol consumption in the evening; mosquito coils at night; lighting a fire and mosquito coils (Sanann et al [121]); people use smoke from fires or from cigarettes outdoors during these evening biting hours to decrease mosquito nuisance; when visiting villages, young people assemble outside at sunset for evening activities (eg, playing volleyball or cards, watching television)	Livelihood activities Recreational activities
Yunnan Province, China: Xu et al (2015) [122]	Questionnaire data on demographics and potential risk factors, including housing condition, local ecology, socioeconomic status, behavior, occupation, activities, travel, malaria awareness and knowledge, and use of malaria prevention measures at both subjects’ home in Yunnan and the locations where they had stayed 1 mo before the date of malaria attack in case patients (retrospective case-control study)	Stayed overnight in Myanmar within 1 mo of the malaria episode and significant associations with lumbering, housing conditions (shelter hits, houses), antimosquito measures, hill zone, proximity to breeding sites, and nearby vegetation	Livelihood activities
Kudat District, Sabah, Malaysia: Barber et al 2013 [47]; Chua et al (2019) [48]; Grigg et al (2017) [30]	Questionnaire data on demographics, behavior, and residential malaria risk factors in a case-control, prospective clinical study	Overnight travel and sleeping outside in the forest or plantation; sleeping under a bed net	Recreational activities; livelihood activities
Ubon Ratchathani Province, Thailand: Lyttlelton (2016) [123]	Qualitative data collection and interviews in malaria outbreak districts covering past and present livelihoods, experience with malaria and programs to alleviate this, and rosewood collection	Overnighting in a national park and forest adjoining Laos and Cambodia in search of endangered tree timber; rosewood secreted through the forest at night to a border crossing with Laos; staying for a week in the forest moving by night and resting by day	Nighttime forest missions undertaken by the poor who have skills in finding, harvesting and portering rosewood.
Tak Province, Thailand: Edwards et al (2019) [44]; Parker et al (2015) [124]	Mixed methods comprising cross-sectional behavior and bed net survey, transect walks observational and entomological collections in villages, hamlets, and forested farm huts.	Slept in the farm huts/forest and bed nets overnight; bathing, exercise/football, cycling/walking, driving motorbike, children playing, smoking, border patrol, conversing/on phone, alcohol drinking, eating, cooking, collecting crickets wood chopping, feeding animals, working, shopping.; most rural villages in area follow a basic calendar revolving around rice paddy cultivation and several other crops (Shoklo Malaria Research Unit [2016] [125]); majority used repellents, followed by wearing of long clothing	Recreational activities Livelihood activities
Khanh Vinh District, Khanh Hoa Province, Vietnam: Edwards et al (2019) [45]; X. N. Xuan (unpublished report) Bac Ai and Ninh Son districts, Ninh Thuan Province: Grietens et al (2012) [126]	Mixed methods comprising (1) cross-sectional behavior and net survey, transect walks, and observational and entomological collections in 3 ecotypes; (2) integration of qualitative data from focused ethnography and quantitative data collected during a large-scale cross-sectional survey for a LLIHN trial (2005–2006)	Slept in farm huts/forest with bed nets or LLIHNs overnight; bathing, listening to radio, conversing, alcohol drinking, eating, cooking, working, walking, other activities Farming (rainy season), hunting, gathering forest products, logging (dry season), entailing overnight stays in rain forest Watching television, singing karaoke, drinking at small bars Approximately half (52%) and 92% slept by 1900 and 2100 h, respectively Irregular forest overnights: almost exclusively farming during rainy season, while other activities (hunting, gathering forest products, logging) are carried out mostly in the dry season, entailing overnights deep in the rain forest.	Recreational activities Livelihood activities Leisure activities Livelihood activities
Xieng-Ngeun and Nane districts, Luang Prabang Province, Lao PDR: Tangena et al 2017 [42]	Mixed methods: triangulating qualitative data from focused ethnography and quantitative data collected during a large-scale cross-sectional survey carried out in the framework of the LLIHN trial Adult mosquito collections in secondary forests, mature and immature rubber plantations, and villages. Rapid participatory rural appraisals and surveys for daily and monthly activities of the rubber workers and villagers.	Visiting secondary forests during rainy season, most frequently during daylight hours (0500 to 1700 h) to collect food, wood, and other commodities; occasional night visits to hunt small animals; night rubber tapping in the rainy season, between 0200 and 0700 h, and latex is collected in the morning from 0700 to 1000 h; from 1700 to 0700 h, most people were usually in the village to cook, clean, and sleep (sleeping time from 2000 h to 0500 h) High ownership (90%) of ITNs in houses; methods used for self-protection against mosquitoes when outdoors are mosquito coils (60%), DEET (35%), wearing long sleeves (7%), and applying lemongrass (2%)	Livelihood and recreational activities; 4 distinct behavioral typologies were identified: (1) villagers that visit the forest during the day, (2) villagers that work in the rubber plantations, (3) migrant workers that live and work in the rubber plantations, and (4) villagers that stay in the village
Maluku, North Maluku, East Nusa Tenggara, West Papua, Papua, Eastern Indonesia: Ipa et al (2020) [127]	Community-based survey (2018 Riset Kesehatan Dasar [Riskesdas]) describing preventive practices at individual and household levels and association with the incidence of malaria among adult (aged >15 y) populations	Used a bed net while sleeping the night before the survey; use of mosquito coils or applied electric antimosquito mats and installation of mosquito window screens (with no identification to night use)	Routine household activities
Mugil and Lemakot areas, Papua New Guinea: Rodriguez-Rodriguez (2019) [50]	Cross-sectional malaria indicator survey; in-depth interviews; focus group discussions	Sleeping times and LLIN use the previous night; use of clothing and footwear; funeral ceremonies; overnight supervision of coconut and cocoa bean drier	Livelihood activities; routine household activities; outdoor hunting and sleeping (during hot nights); recreational and religious activities; large-scale social festivals
Central Island Province, Solomon Islands Pollard et al 2020) [53]	Daily movement diaries, interviews, and direct observations	Overnight people movement: 34% of participants (n = 29) spent ≥1 night away from the village with frequent overnight travel to another village (59%) followed by a town or city (24%) and employment on ships (14%)	Overall people movement was designated as being within 1 of 4 nested categories, of increasing scale: inside the house, peridomestic area around the house (including veranda and external kitchen building), the residential village, and all areas beyond the village

Abbreviations: DEET, N,N-diethyl-meta-toluamide; ITNs, insecticide-treated bed nets; LLIHN, long-lasting insecticidal hammock net; LLIN, long-lasting insecticidal net; PDR, People’s Democratic Republic.

Methods used in these 10 studies document and characterize human behavior included participant observations, in-depth interviews, rapid participatory rural appraisals, structured observation surveys, questionnaires, focus group discussions, mixed methods, transect walks, triangulation of qualitative and quantitative data, daily movement diaries, and Global Positioning System trackers. Some studies often looked at specific nighttime or daytime activities, as well as the impact of these activities on use of malaria prevention tools. Risk factors can include occupational exposures and other behaviors outside of households (eg, forest-going, farming, and cooking). Mobility of individuals and/or population groups may greatly vary (daily, weekly, seasonally), which in turn may affect the impact and effectiveness of vector control interventions, and consequently malaria risk.

Given the paucity of literature on observations of nighttime activities, we provided some informal field observations by malaria veterans below. Informal field observation 1 is from a Khmer refugee camp, Thailand-Cambodia border (1980–1981):

… when I worked in the “Kampot” Khmer refugee camp at Pong Nam Ron, right across the border from Pailin, 1980–81, I was hired to control a malaria outbreak in the camp, 17 000 souls, by setting up ULV sprayer coverage, which I did. But I also did some epidemiology and found that the assumption of domestic transmission in the camp was wrong. First of all, most of the cases were adult men, even though everyone slept in bednets in 3-sided houses, went to bed at 9pm (curfew) and got up at the same time in the morning. Second, the hypothesized vector was dirus, flying into the camp from a nearby forest, however, there were no identifiable breeding sites, and nighttime collections caught no dirus. Third, and definitive, when I went out to find and followup on patients for recrudescence, they could not be found, but/and when my Khmer microscopist when out, he reported that the patients (mostly adult male) were not in the camp, which directly contradicted the firm assertions by camp officials that no one left the camp, due to barbed wire fences and armed Thai soldiers. The Khmer left the camp at night, by truck, to work in Thai fruit orchards, and came back to camp when they got malaria. Another example of non-traditional transmission, ie, not in the home. This was a blind spot for all of us for many years. I could have had a rich and full career talking about mobile and migrant malaria, starting in the 1980s, if I had recognized it at the time (Steven Bjorge, personal communication, July 15, 2020).

Informal field observation 2 was from Hilltribe Village in Mae Hong Son (2000):

Malaria transmission was occurring among school children staying in a boarding school in a forested community. It was determined that the infected children had been regularly watching TV soap operas in the (unscreened) library after dinner from 8–9 p.m., prior to sleeping in a screened dormitory (Jim Hopkins, personal communication, July 15, 2020).

Informal field observation 3 was from Karen Hilltribe Village in Tha Song Yang, Tak Province (2005):

As a member of the Global Fund Round 2 Malaria Evaluation Team, I visited a village of bamboo houses with thatched leaf roofs on the Thailand-Myanmar border with the highest incidence of MDR falciparum malaria in Thailand. ITN coverage was more than 100% and the village had a full-time malaria volunteer post with RDTs and radical treatment drugs. Each house had a bamboo pole antenna, a solar panel, and a car battery connected to a television. A quick survey indicated that villagers used the solar-powered battery to watch television for 2–3 hours from 6–9 p.m. through the prime time soap opera, prior to setting up their ITNs for sleeping (Jim Hopkins, personal communication, July 15, 2020).

Informal field observation 4 was from market places in Southern Palawan (2019):

Southern Palawan with hilly and forested terrain is the last hold-out of endemic malaria in the Philippines. Transmission persists despite LLINs, IRS and curative services. As elsewhere in Southeast Asia, this has been ascribed to deep forest farming from unprotected dwellings, and additional LLINs have been distributed for those situations. It is increasingly accepted that there is also some malaria risk at marketplaces, where whole families often stay overnight, the children enjoying TV shows and videogames thanks to generators. Some representatives of indigenous people’s organizations have opined that the malaria risk at those market-places could be higher than in the home or at the deep forest plot-huts. One barangay captain (elected community leader) had just decided, last time I was there, in November 2019, to close the market at 6 pm to prevent malaria. I pointed out that people would then be exposed when walking home, and advised to close at 4 pm or not close but put emphasis on the giant collective insecticide-treated nets, which were being made available for those settings by the national malaria programme and its partners (Allan Schapira, personal communication, Jul 17, 2020).

Informal field observation 5 was from touch points in forested areas of Pursat, Cambodia (2017):

As many people who live in or near the forest do not have access to electricity, they often gather together with family or friends in the evening. There are no days off, so each day is similar, with people often being physically exhausted after working all day in the farm or forest. They often want to just chat with friends over dinner, and live or visit in structures which are not closed, do not sit inside nets while eating dinner or visiting friends, and do not wear long sleeves due to the heat. They sometimes gather at a nearby house or village if someone has a generator to watch TV, however listening to the radio is the most common activity especially among the older generations. The most popular and available vector control products (other than ITNs) are mosquito coils and insecticidal sprays, although they often do not have enough money to use them all the time (John Hustedt, personal communication, July 21, 2020).

### Review and Characteristics of Nighttime Activities

We included 10 studies (with multiple authors from companion articles) of some descriptions of evening activities occurring during times when local malaria vectors are active (Table 3). These studies identified activities taking place in the peridomestic setting, which are quite similar to those in the study by Monroe et al [34]: for example, activities “inside and directly outside of the home as well as away from home throughout or part of the night.” Similar patterns regarding “routine household chores and entertainment occurring in the evening hours before bed, routine livelihood activities that lasted throughout the night such as security and fishing, and large-scale sociocultural events, such as funerals that might last throughout the night” [34] were also seen in the Asia-Pacific region.

### Influence of Insecticides and Behavior of Vectors

One of the major threats for control of RMT is the development and spread of insecticide resistance (IR) in vector populations expressed as either physiological or behavioral resistance [128–130]. Care should be exercised not to overstate the risk of insecticide resistance in forest settings where selection pressure is almost entirely absent. Many NMCPs are doing active monitoring for insecticide phenotypes to insecticides used in control programs, usually through limited sentinel site surveillance rather than in response to increased malaria burden, so it is unlikely that resistance will be detected at an early stage. Consequently, funding partners with limited understanding of entomological issues (and understandably) tend to fund what they know: “vector bionomics and insecticide resistance in sentinel sites,” rather than what would be most useful to the program in a resource limited environment [59]. In addition, data to determine actual mechanisms (ie, metabolic and target-site) responsible for resistance are rarely collected with only 14% of Asia-Pacific countries evaluating insecticide resistance mechanisms; [36]. Because this is an essential prerequisite for maintaining intervention effectiveness by enabling more proactive decision making management for IR management [131], and despite “a high prevalence of countries monitoring IR phenotypes, not all countries monitoring IR have strategic frameworks for responding to the data (eg, IR management plans) and among those with a management plan, not all report using their data as a basis for maintaining or selecting alternative insecticides for programme use” [36].

The presence of insecticides can not only act to suppress susceptible vector populations as toxicants but can also modify normal behavioral responses, such as host seeking and biting activity regardless of susceptibility status (ie, physiological resistance). So-called behavioral resistance, as reported in the literature [50, 78, 132–134], is likely more a reflection of a stimulus-response (eg, avoidance or deterrence) to the presence of a chemical operating as contact locomotor excitation agent and/or as spatial repellent [135–138]. In particular, DDT and many pyrethroid class chemicals used to treat bed nets and IRS possess such behavioral excitorepellency responses. In some instances, they has been reported to have “changed” (or modified) the temporal and spatial blood feeding behavior of the vector population, typically a “shift” to earlier evening biting hours and a higher proportion of the population feeding outdoors away from residual insecticides present indoors (ie, sprayed walls or ITNs).

This is the case for *Anopheles farauti* in Papua New Guinea and Solomon Islands and *A. dirus* in Thailand, which impose an enormous challenge for malaria control owing to behavioral avoidance of insecticide-treated surfaces (Table 4). Interestingly, the widespread use of IRS begun in the 1960s has nearly eliminated *Anopheles koliensis* and *A. punctulatus*, species that are mainly endophagic, in the Solomon Islands [133], with replacement by *Anopheles hinesorum*, a relatively poor vector, if not nonvector [139], and *A. farauti*, a more exophagic and early-biting mosquito, often present in high densities [7, 133].

**Table 4.** Review of the Insecticide Susceptibility and Vector Behavior Based on Vector Control Measures in Selected Countries^a^

Location and References	Insecticide Susceptibility		Shifts in Species Frequency and Behavioral Shifts to Early Biting, Outdoor Feeding
	Primary Vector Species^b^	Secondary Vector Species^b^	
Usino-Bundi, North Coast and Ramu (Madang Province), Drekikir (East Sepik Province), Lorengau (Manus Province), Papua New Guinea: Keven et al (2010) [140]; Henry-Halldin et al (2012) [141]c	*Anopheles farauti* s.l., *Anopheles punctulatus* susceptible (phenotypic) to 0.05% lambda-cyhalothrin and deltamethrin LLIN (55 mg/m^2^)	*Anopheles hinesorum* susceptible (phenotypic) to 0.05% lambda-cyhalothrin and deltamethrin LLIN (55 mg/m^2^).	*A. farauti* s.l. developed behavioral resistance to avoid contact with DDT (Spencer et al [1974] [169]); after ITN distribution, biting cycles of both *A. farauti* and *Anopheles koliensis* shifted from a postmidnight peak to an earlier premidnight peak in a coastal village (Charlwood and Graves [1987] [132]); in coastal and inland foothills, shifts in mosquito biting to earlier hours after the first LLIN distribution (the peak exposure time to infectious bites shifted from after 2100 h in 2008 to between 1800 and 1900 h in 2011, resulting in decreased protection against mosquito bites (Reimer et al [2016] [78], Thomsen et al [2016] [134], and Rodriguez et al [2019] [51])
Madang, Milne Bay, East Sepik, and East New Britain provinces, Papua New Guinea: Koimbu et al (2018) [142]	*A. farauti, A. koliensis, A. punctulatus* susceptible (phenotypic) to 0.05% deltamethrin, 0.05% lambda-cyhalothrin, 4% DDT	ND	
Western, Temotu, Central, Choiseul, Malaita, Guadalcanal provinces, Solomon Islands: Quiñones et al (2015) [143]	*A. farauti* susceptible (phenotypic) to 0.05% deltamethrin (2014); moderate resistance to 0.05% lambda-cyhalothrin (Malaita and Central), 0.5% permethrin (Central and Guadalcanal) and 0.05% deltamethrin (Guadalcanal)	ND	IRS in the 1960s nearly eliminated major malaria vectors *A. koliensis* and *A. punctulatus*, mainly endophagic and late-evening biters (Taylor [1975] [133]); a behavioral shift in *A. farauti* s.l. toward earlier feeding with a higher proportion of feeds occurring outdoors after IRS (DDT) in the 1960s–1970s (Taylor [133]) and after 1992 (Over et al [2004] [159])
5 Districts in Ubon Ratchathani Province, Thailand: Sumarnrote et al (2017) [144] Bo Rai District, Trat Province, Thailand: Pimnon and Bhumiratana (2018) [118] Country-wide site selections, Thailand: Van Bortel et al (2008) [145]	*Anopheles dirus* s.l., *Anopheles maculatus* s.l.. susceptible (phenotypic) to 0.05% deltamethrin (insufficient numbers) *A. dirus* s.l. susceptible (phenotypic) to 0.05% deltamethrin and 0.9% bifenthrin (insufficient numbers) *Anopheles epiroticus, Anopheles minimus* s.l. susceptible (phenotypic) to 0.75% permethrin; *A. epiroticus* susceptible to 4% DDT (insufficient numbers)	*Anopheles barbirostris* s.l. susceptible (phenotypic) to 0.05% deltamethrin, resistant to 4% DDT; *Anopheles nivipes, Anopheles philippinensis* susceptible to 4% DDT, 0.05% deltamethrin, and 0.75% permethrin; PBO increased mortality rate with deltamethrin and permethrin in pyrethroid-resistant *Anopheles hyrcanus* s.l.; none of the sequenced specimens showed *kdr* gene1014F or L1014S mutation *Anopheles campestris* resistant to 0.05% deltamethrin and tolerant to 0.09% bifenthrin *Anopheles scanloni* susceptible to 4% DDT and 0.75% permethrin	IRS resulted in a higher proportional decrease of *A. dirus* s.l. as compared with *A. minimus* s.l. (Ismail et al; [1975] [113] and Pimnon and Bhumiratana [118]). Widespread use of IRS resulted in modified behavior of *Anopheles minimus* s.l. (Nustsathapana et al [1986] [113]), which might reflect a species shift from *A. minimus* to *Anopheles harrisoni*, as also observed in Vietnam, the result of widespread ITN use (Garros et al [2005] [158] and Edwards et al [2019] [45]) Bifenthrin IRS did not reduce indoor densities relative to outdoor *A. campestris*, except at 6 mo after IRS (Pimnon and Bhumiratana [118])
3 sites, Cambodia: Van Bortel et al (2008) [145]	*A. dirus* s.l., *A. minimus* s.l. susceptible (phenotypic) to 0.75% permethrin; suspected resistance to 4% DDT (insufficient numbers); *A. epiroticus* susceptible (phenotypic) to 0.75% permethrin; possible resistance to 0.05% deltamethrin.		Before widespread ITN use, 29% of the bites occurred before sleeping time in villages and forest plots [40]
Vietnam: Van Bortel et al (2008) [145]	*A. dirus* s.l. susceptible (phenotypic) to 0.75% permethrin; possible resistance to 0.05% alpha-cypermethrin; 1 *A. dirus.* population and 5 *A. minimus* s.l. populations resistant to lambda-cyhalothrin, 3 of 6 *A. minimus* populations with possible resistance to 0.5% etofenprox		Widespread ITN use associated with a species shift from predominately *A. minimus* to *A. harrisoni* (Garros et al [158]), and disappearance of *A. minimus* in Khanh Phu Commune (Marchand, personal communication, March 6, 2019)
Lao PDR: Van Bortel et al (2008) [145]; Marcombe et al (2018) [146]	*A. minimus* s.l. susceptible (phenotypic) to 0.75% permethrin (insufficient numbers); no resistance to pyrethroids was detected; *A. dirus* s.l., *A. minimus* s.l. and *A. maculatus* s.l. with suspected resistance to 0.75% permethrin in Phongsaly and Luang Prabang provinces; suspected resistance to 45 DDT from Saravane and Attapeu provinces	*A. nivipes* and *A. philippinensis* with no resistance to pyrethroids and high resistance to 4% DDT in Khammouane Province	ND
China: Cui et al 2006 [147]; Suman et al (2013) [148]; Chang et al (2014) [149]; Fang et al (2019) [150]; Chen et al (2019) [151]	*Anopheles sinensis* with low to moderate resistance (genotypic) to permethrin and deltamethrin; confirmed resistance to DDT and malathion from the malaria-endemic areas		ITN use decreased the endophilic and anthropophilic *Anopheles lesteri* (*Anopheles anthropophagus*) and *A. minimus* s.l. compared with the more exophagic/zoophilic *A. sinensis*

Abbreviations: IRS, indoor residual spray; ITN, insecticide-treated bed net; LLIN, long-lasting insecticidal net; ND, not done; PBO, piperonyl butoxide; PDR, People’s Democratic Republic; s.l., sensu lato.

^a^Unpublished reports can accessed using the World Health Organization interactive mapping tool (https://www.who.int/malaria/news/2017/malaria-threats-map/en/).

^b^Noted that “s.l.” is added to the species name when referring to the species complex (*A. minimus* s.l*., A. dirus* s.l.). In the absence of s.l., the species (taxon) is indicated (eg, *A. minimus*, *A. dirus*).

^c^Including Manus, Morobe, West Sepik, and Western Highlands provinces.

Currently, the primary malaria vectors in Papua New Guinea appear to remain susceptible to pyrethroids [56, 57]; however, despite mass distribution of ITNs throughout malaria-endemic areas of the country, transmission continues to persist at high levels. Entomological studies conducted by NMCP in partnership with Rotary Against Malaria and the Papua New Guinea Institute of Medical Research have revealed that very low bioefficacy of LLINs procured from 2014 onward [110] may have been among the contributory factors in the malaria resurgence, including poor adherence to case management [152]. In addition to outdoor blood feeding, persistent transmission may be due, in part, to the more generalist, broader range of host selection by the primary vector species. As a species group, the Punctulatus assemblage is typically opportunistic in feeding (varying anthropophilic to zoophilic), based on host availability and ITN usage [134]. Although vectors readily feed on humans, opportunistic host selection, partly a function of relative host availability and a propensity for using alternative animal hosts (eg, domesticated pigs, dogs) has been well described [153, 154]. 

In Papua New Guinea, it was reported that hosts were not selected in proportion to their abundance, but rather were either underselected or overselected by the mosquitoes [133]. Four species, *A. farauti* s.s., *A. punctulatus* s.s., *A. farauti* no. 4 and *Anopheles longirostris,* overselected humans in villages with low LLIN usage but overselected pigs in villages with high LLIN usage. *A. koliensis* consistently overselected humans despite high LLIN usage, and *Anopheles bancroftii* overselected swine [133]. A lack of selectivity for human blood allows these vectors to escape exposure or greatly minimize contact with indoor treated surfaces or nets entirely, thus precluding sufficient selection pressure promoting resistance. A recent review concluded that zoophagic mosquitoes “with moderate vectorial capacity often respond poorly to LLIN or IRS interventions because the technologies are designed to target the stereotypical behaviors of the smaller number of more potent human-specialized species that mediate most, but by no means all, of the global malaria burden” [8]. Killing or reducing mean survival in these mosquitoes with livestock-based interventions, such as topical or systemic insecticides or endectocides ,could play a complementary role in reducing RMT [8, 155, 156].

The Punctulatus group vector species commonly exist in sympatry throughout most of the malaria-endemic areas of Papua New Guinea [22, 24, 40, 41, 44, 55–57]. While ITNs may affect the anthropophilic species (eg, *A. koliensis*), transmission is still sustained by the more opportunistic and behaviorally “plastic” species that will feed on other animals as availability permits. This condition, when combined with increased outdoor and early-evening biting observed in some vector population of Papua New Guinea ([78, 134]), presents a challenge to the ITN program in Papua New Guinea, as well as the rest of the Pacific region, where the more opportunistic species *A. farauti* s.s. and *A. punctulatus* are the primary regional vectors. By maintaining high population size, the proportion that feed on humans outdoors can sustain residual transmission despite high ITN usage in the village [157].

In central Vietnam, the *A. minimus* population has virtually disappeared after the introduction of ITN [158] and remained absent for the next 18 years (Ron Marchand, unpublished data). In Assam, northeastern India, *A. minimus* mosquitoes were not seen resting inside human dwellings after an initial 3 years of continuous ITN distribution [160]. The ITN-based intervention not only deterred entry of *A. minimus* species, but also served as personal guard against infective mosquito bites corroborated by data on human mosquito landing catches and declining trends of malaria transmission [161]. The use of public health insecticides in Nepal eliminated *A. minimus* s.l. [162, 163] and significantly reduced populations in the Thailand peninsula and central plains, although they did remain abundant in hilly forested areas [164, 165]. In malaria-endemic areas of China, ITN use resulted in a higher decrease in the endophilic and anthropophilic *Anopheles lesteri* (*Anopheles anthropophagus*) [166] and *A. minimus* s.l. [167] relative to more exophagic and zoophilic *Anopheles sinensis*, a species that also shows high levels of multiple insecticide resistance. (Table 4).

Vectors can have a repertoire of behavioral actions to external stimuli that can effectively avoid contact with insecticides [130, 168], which may be a result of innate, preexisting variability (plasticity or resilience), resulting from an instantaneous stimulus-response in a species or particular population or possibly the genetic selection over time of a population to avoid the real or potential presence of inimical chemicals in the environment that expresses an evolutionary advantage (“resistance”). For example, behavioral resistance can be expressed as changes in either the location where vectors seek blood meals (ie, shifting from indoor to outdoor biting) or the time when blood meals are taken (ie, feeding earlier in the evening or in the early morning hours when people are outside their houses and not protected by ITNs or IRS) [36]. Examples of observed shifts in species population behavior (eg, the near-elimination of *A. koliensis* and *A. farauti* in Solomon Islands; decrease of *A. dirus* s.l. relative to *A. minimus* s.l.; species shift from *A. minimus* to *Anopheles harrisoni* in Thailand; rebound of *Anopheles fluviatilis* s.l. and *Anopheles maculatus* s.l. after IRS in Nepal), shifts to outdoor biting or early biting periods (eg, *A. farauti* and *A. koliensis* in Papua New Guinea and Solomon Islands; *A. dirus* and *A. minimus* in Thailand and Vietnam), shifts to zoophily (*A. farauti* in Papua New Guinea, *A. minimus* in Thailand, *Anopheles culicifacies* in India) or to exophily (*A. farauti* in Solomon Islands, *A. culicifacies* in India) are reported in the Asia-Pacific region associated with the use of routine ITNs and IRS [7].

Given the significant limitations in the scope and methods of historical literature and sparse data in response to LLINs and IRS, it is suggested that findings accurately describe behaviorally resilient rather than resistant mosquito taxa, which have always exhibited evasive traits [138]. Because the precise mechanisms driving these genetical shifts have not yet been fully elucidated, the vast majority of behavioral response variation to stimuli and conditions is due to inherent “plasticity” in the mosquito. Thus, it is inappropriate to infer selection of new behavior patterns when mosquitoes show an inherent plasticity in feeding after being frustrated in accessing their hosts [170]. More research on methods, the natural heterogeneity of vector populations, and the role of heritable traits in behavioral resistance are needed to understand better the spectrum of changes induced by intensive insecticide use for future policy discussions [170].

Because “[m]ost NMCPs are unlikely to detect the emergence of behavioral resistance with their existing vector surveillance programs as only 31% of countries monitor the indoor-outdoor biting ratio with 34% tracking changes in peak biting time, the 2 most common expressions of behavior resistance” [36], it is important to know which humans to target and when and where to target humans who are exposed to mosquito bites. For example, survey data on human behavior and high-risk populations can be analyzed together with data on vector bionomics and intervention efficacy, which may help determine gaps in protection and local drivers of transmission, including drivers of residual transmission. While there is a growing research agenda on this topic [44, 45, 171] program-oriented methods are currently available for national malaria programs to consider using [119]. Integrating vector behavior and insecticide resistance data with human behavioral observations will demonstrate where and when people are exposed to mosquito bites, as well as potential gaps in protection indicating that supplemental tools may be needed. The effects of insecticide resistance and behavioral responses of vectors on malaria transmission can vary, depending on a range of different epidemiological factors, being species and location specific [172]. Regardless of the mechanism involved, movement of vector populations to early biting times, when more people are active outdoors, is an important consideration for applying control measures and an obstacle for achieving elimination.

Given the complete susceptibility (100% mortality rate) of field-collected populations of *A. harrisoni* (Minimus complex species) and *A. dirus*.to transfluthrin in Thailand, [173], there is potential for deploying highly volatile pyrethroids in situations where conventional control methods are not readily accessible and where repellent devices may complement other currently deployed methods of protection (eg, topical repellents, LLIN, and IRS) [174, 175]. Sangoro et al [175] demonstrated that using transfluthrin-treated chairs and ribbons reduced outdoor-biting malaria vectors in peridomestic spaces, and also elicited significant mortality among pyrethroid-resistant field-caught malaria vectors. The use sandals treated with 0.05 g of transfluthrin sandals reduced exposure to *Anopheles gambiae* s.l. landings by at least 50% and 40% against *Culex* mosquitoes in the field. From the public health impact, the combined use of metofluthrin-impregnated spatial repellent devices and LLINs (Olyset Plus) reduced the infection rate in children as well as the number of pyrethroid-resistant vector mosquitoes in malaria-endemic villages in southeastern Malawi [176].

## DISCUSSION

The current review identified several areas of importance related to RMT. The first highlights the inefficiency of current measurement tools, which produce data often confounded by human and/or mosquito behavior and results in a misclassification of “residual” transmission. In situations where malaria is considered residual, as in Vietnam and Cambodia, primarily because of the very high population coverage of ITNs, the persistence of malaria transmission has moved from villages to farm plots and forested areas, often involving primary and/or secondary vector species [44, 45, 100]. The lack of high-quality coverage data on ITN access and use at subnational levels in many countries presents a significant challenge and hampers a full understanding of RMT dynamics. Where such data exists at the district level or at lower administrative levels (eg, the health facility or village level), not only does the deployment of ITNs require new approaches, but other vector control tools are also needed. The second area relates to when (time of day) and where (indoors vs outdoors) people are exposed to malaria vectors, which was evaluated in just 5 studies (in Thailand, Vietnam, Cambodia, Solomon Islands, and Papua New Guinea) [42, 50, 53, 121, 126].

The third aspect is night activities that may increase people’s contact with malaria vectors. Where data is available, a good understanding of human behavior is crucial for targeting context-appropriate vector control interventions across specific settings. Given the inherent heterogeneity of malaria transmission across different landscapes, it is imperative that data and information be “local” to identify the relative importance of specific activity categories and target groups based on the entomological, human behavioral, and epidemiological context. To do so requires a highly coordinated, interdisciplinary approach involving input from NMCP managers, social scientists, and public health entomologists to help prioritize and interpret data on the access to and use of ITNs and to clarify the gaps in protection.

The fourth aspect is gender-related vulnerabilities and barriers for accessing malaria services that fuel persistent malaria transmission from cross-border or intraborder mobility of illegal migrants and indigenous populations. The available data suggest that outside forest communities, where entire families resides inside the forest, it is predominately younger males who are more frequently accessing the forest [42, 53, 177]. The acceptability and use of ITNs are strongly linked to cultural norms of sleeping patterns, in which sex and age play important roles. In Shan state, Myanmar, young children of ethnic groups and Chinese inhabitants shared nets with adults, presumably with their mothers, and are therefore protected by their mothers’ ITNs, if available [178]. However, a follow-up study in 2016 covering 32 052 people, including 1514 forest-goers from every state and region (except Chin State) in Myanmar, showed that age and sex had little effect on net or ITN/LLIN use [59]. 

Another study highlighted poor ownership and use of ITNs among migrants in the Regional Artemisinin Initiative project areas of Myanmar, including identified barriers to their ownership and use [179]. In mapping the population, there is a need to consider sex-related behavioral differences, especially for the reproductive age group, because sharing and sleeping under a single net together is inappropriate [179]. For instance, this would apply for implementing behavior change interventions in migrant plantation workers [180]. An example from Nigeria showing sex disparity in ITN use, with males less likely to use ITNs (particularly in those aged 15–25 years), illustrates the need for age-sensitive and gender-sensitive messaging during a universal distribution campaign to ensure that males benefit equally from such communications and activities [181], which may be applicable in the Asia-Pacific region [182]. 

It is essential that social and gender determinants of ITN ownership and usage be explored in high-risk communities, and health communication messages should stress the need for everyone entering the forest or in nearby areas with malaria transmission to sleep under ITNs. The Malaria Matchbox [108] is a useful tool for program managers and implementers to identify barriers that people encounter in trying to access and use healthcare services, particularly those related to malaria prevention and care. Identifying those barriers, whether sociocultural, financial, physical, or related to gender norms, is an essential step to match people’s specific needs to responses that are person centered, rights based, and gender responsive [108].

In the current review, various activity categories (Table 3) are provided that serve as a useful framework for informing context-specific research on the relative importance of these activities that can drive locally appropriate interventions. Incorporating this framework and appropriate methods with the Entomological Surveillance Planning Tool (ESPT) [117] is the next step to develop standardized approaches to guide programmatically relevant data collection on nighttime and daytime activities and categories to interpret ITN use and access information [119].

This review is limited by the possibility that studies that would have met inclusion criteria were not identified in the review process. Particularly, it did not consider factors (or possible “drivers” of transmission) that could influence transmission dynamics, such as changes in malaria receptivity and/or importation risk (ie, vulnerability), including new land development, population movement associated with seasonal agricultural pursuits, periodic political, cultural or religious events, civil unrest, internally displaced populations, environmental parameters such as mean and/or total rainfall by location, and accessibility to malaria diagnosis and treatment. For example, from 2006 to 2016 in Cambodia, the number of confirmed malaria cases decreased 84% from 143 758 to 23 492 [177], while from 2000 to 2014 the amount of dense forest decreased from 35% of land cover to 16% [183]. 

Geographers have noted that the reduction in forest is directly related to the surge in economic land concessions, and that the annual rate of forest loss was between 29% and 105% higher in land concessions than in comparable land areas outside concessions [184]. This probably results in lower species heterogeneity in rubber monoculture plantations than in secondary forests, which provide a higher diversity of aquatic habitats and consequently a higher heterogeneity of mosquito species [185–187]. While all important topics to consider, they were outside the scope of this focused objective. However, with exclusion of population movement as a search term and inclusion criterion, it is possible that relevant articles could have been missed. Nonetheless, a comprehensive and structured process was used.

In addition to identified constraints and limitations of available data (published or otherwise), it was not possible to compare study findings directly owing to differences in study design and methods. However, the general conclusions of the studies in this review unequivocally suggest that a majority of malaria vector biting occurs outdoors during early evening hours, before people retire indoors, followed by indoor transmission for unprotected individuals not using a bed net (treated or not). This is evident even in contexts where unadjusted human biting rates are higher outdoors than indoors. However, when viewing ITN users, we estimated that roughly half of the malaria exposure likely occurred outdoors in some settings, indicating a clear gap in protection.

Furthermore, estimates of the proportion of human exposure to Pacific and Southeast Asian malaria vector populations that occurs indoors for both unprotected residents (π _h,i_) and users of LLINs (π _h,i,n_) were consistently similar in Solomon Islands (π _h,i_, 0.56 [π _h,i,n_, 0.07] for *A. farauti*) and Myanmar (0.41, 0.48, and 0.54 [0.07, 0.04, 0.06, and 0.7] for *Anopheles epiroticus, Anopheles subpictus,* and *Anopheles annularis*, respectively) [7]. While *A. dirus* in GMS can exhibit similarly stereotypical nocturnal, indoor-feeding behavior, this is unusual among vectors found in Lao PDR (π _h,I_, 0.91 [π _h,i,n_, 0.40] for *A. dirus*) [7] and Thailand (1.46 and 5.39 [0.09 and 0.34] for *A. maculatus* and *A. minimus*, respectively) [44]. These vectors exhibited an increased tendency for early outdoor biting after implementation of IRS, resulting in nonuniform exposure [113–115].

One of the most relevant indicators for understanding RMT is the protective efficacy of ITNs, defined as the proportion of human exposure to malaria vectors prevented by ITN use out of total exposure (ie, compared with a nonuser) [120]. Protective efficacy, which is the overall reduction in nightly biting rate for an ITN user compared to a nonuser, ranged from 88% to 93% in 2 settings where ITN coverage was suboptimal (Lao PDR and Thailand), with even lower estimates (<50%) for settings beset with primarily exophagic and/or opportunistic malaria vectors despite high ITN coverage (Myanmar) [46, 75]. Understandably, the fraction of exposure occurring outdoors and indoors during nonsleeping hours poses a significant obstacle to malaria control and elimination efforts [188].

The importance of human behavior for understanding malaria transmission dynamics cannot be underestimated, and yet, even with this commonly acknowledged link with persistent malaria, relatively few studies were identified that included specific human activities as risk factors. For those studies that have identified human factors, different methodological approaches were used across studies, greatly limiting the comparisons for this review.

Here we have identified gaps/deficiencies that have been addressed by ESPT guidelines: (1) standard operating procedures and methods to best estimate risk exposure away from the peridomestic setting; (2) a standard analytical approach to measure human-vector interaction, which should account for outdoor sleeping as well as segments of the population that may spend most or all of the night away from home; and (3) collection of human and vector data close in time and location, and across time points (longitudinally), to reflect changes in vector and or human behavior between seasons as proposed by Monroe et al [120] and Killeen et al [189, 190]. In rural agrarian and swidden agriculture, there is a need to develop location-specific seasonal agricultural or forest calendars to reflect normal activities during the year [125].

Our review identified the ESPT as a standard approach to validate the estimates used for collecting human behavioral and entomological data—an important next step for developing a more uniform understanding of RMT and risk factors in general for those vulnerable populations subjected to persistent malaria. Since linking human activities with entomological parameters of transmission is generally lacking, these parallel domains need to be carefully integrated if we are to make sound epidemiological sense of the transmission dynamics at work. Finally, only with a better understanding of the gaps in protection can other appropriate vector control interventions be introduced or developed, over time and across settings, to address these challenges. To do so must take into account the health systems enablers, such as capacity to maintain supply chains of necessary commodities, maintain high-quality, timely, and responsive heath information systems and develop human resource capabilities as well as adaptive capacity at the district to community level. This type of data will be needed to ensure access to and encourage use of LLINs and vector control products among at risk populations.

In conclusion, perhaps the most important finding of this review pertains to the current state of the published research literature, which was limited at best. Standardized procedures and methods to estimate human exposure to mosquitoes indoors for unprotected residents at high vector control coverage within and beyond the peridomestic setting was not provided in 8 of the 14 survey-based studies included in this review. This omission rendered it impossible to reliably interpret the relative importance of the findings in the respective studies. Reliable interpretation of entomological and human behavioral data was often undermined by inadequate description of the study design and methods. Examples included the frequent failure to report how data were obtained (eg, in response to a structured checklist or guidelines provided by ESPT). The omissions described above likely reflect that the focus of this review (reasons for not integrating entomological and human behaviors as reported by study participants) was rarely a primary focus of RMT studies as identified by the search method. On the other hand, well-documented descriptive analysis of nighttime human activities and use of preventive measures were reported in 16 studies among 8 countries of interest: Cambodia (3 studies) [19, 62, 121], China (1 study) [122], Indonesia (1 study) [127], Lao (1 study) [42], Malaysia (3 studies) [30, 47, 48], Papua New Guinea (1 study) [50], Solomon Islands (1 study) [53], Thailand (3 studies) [44, 123, 124], and Vietnam (2 studies) [45, 126].

Taken together, the omission of important data and information in many of the survey findings, the lack of a clearer understanding of RMT concept, and the dearth of dedicated mixed methods (eg, quantitative and especially qualitative investigations) seriously compromised the findings cited in this review. The seemingly clear patterns evident in the reviewed data and the recommended interventions should, therefore, be considered cautiously and highly tentative until greater numbers of well-designed studies are available in the literature. The current evidence-based review is not sufficient in scope or quality to reliably inform personal protection or preventative promoting interventions or broader community campaigns targeted at individuals who possess but do not (reliably) use mosquito nets or long-lasting hammock nets.

## Notes

***Acknowledgments*.** This paper is dedicated to the memory of Dr. Michael Bangs who took a special interest in this study and shared his experience & knowledge from the Asia Pacific region. Mike wrote the section on “influence of insecticides and behavior of vectors”, reviewed and edited drafts of the paper, and approved the submitted version. Mike’s contribution to the fight against infectious disease, and malaria in particular, especially through his work with NAMRU, International SOS, and many others cannot be overstated. From his ground-breaking work as an entomologist to high-level technical analysis, he placed Asia in the epicentre of this fight. He will be greatly missed. We are grateful to the following individuals for providing valuable input, including unpublished reports and advice in the development of this manuscript: Y Tanrang (Sabah Department of Health, Kementerian Kesihatan Malaysia), Bouasy Hongvanthong (Centre of Malariology, Parasitology and Entomology, Ministry of Health, Lao People’s Democratic Republic, Amelie Vantaux (Institut Pasteur Cambodge), Steven Bjorge, James Hopkins, Lucas Nene, and Allan Schapira. We also thank Michael Macdonald, Sean Hewitt, and Allan Schapira for their constructive comments on earlier drafts.

***Financial support.*** This work was supported by the WHO Special Programme for Research and Training in Tropical Diseases in Geneva.

***Supplement sponsorship***. The supplement is sponsored by TDR, the Special Programme for Research and Training in Tropical Diseases, based at the World Health Organization.

***Potential conflicts of interest.*** All authors: No reported conflicts. All authors have submitted the ICMJE Form for Disclosure of Potential Conflicts of Interest. Conflicts that the editors consider relevant to the content of the manuscript have been disclosed.

## References

[1] World Health Organization. Regional action plan 2017–2030: towards a malaria-free South-East Asia Region. 2017. https://apps.who.int/iris/handle/10665/272389. Accessed Jun 24, 2020.

[2] World Health Organization. Regional Office for the Western Pacific. Regional action framework for malaria control and elimination in the Western Pacific: 2016-2020. Manila: WHO Regional Office for the Western Pacific. 2017. https://apps.who.int/iris/handle/10665/255474. Accessed 24 June 2020.

[3] Bhatt S , Weiss DJ, Cameron E, et al The effect of malaria control on *Plasmodium falciparum* in Africa between 2000 and 2015. Nature 2015; 536:207–11. 10.1038/nature15535 PMC4820050 26375008

[4] Hemingway J . Malaria: fifteen years of interventions. Nature 2015; 526:198–9. 26450051 10.1038/526198a

[5] World Health Organization. World malaria report 2019. Geneva, Switzerland: World Health Organization, 2019.

[6] The malERA Consultative Group on Vector Control. A research agenda for malaria eradication: vector control. PLoS Med 2011; 8:e1000401. 21311587 10.1371/journal.pmed.1000401 PMC3026704

[7] Durnez L , Coosemans M: Residual transmission of malaria: an old issue for new approaches. In Anopheles mosquitoes - New insights into malaria vectors (Manguin S. Editor. Rijeka: Intech; 2013:671-704. https://www.intechopen.com/books/anopheles-mosquitoes-new-insights-into-malaria-vectors/residual-transmission-of-malaria-an-old-issue-for-new-approaches. Accessed May 10, 2020.

[8] Killeen GF . Characterizing, controlling and eliminating residual malaria transmission. Malar J 2014; 13:330. 25149656 10.1186/1475-2875-13-330 PMC4159526

[9] World Health Organization. Guidance note on the control of residual malaria parasite transmission. 2014. https://www.who.int/malaria/publications/atoz/guidance-control-residual-transmission/en/. Accessed 19 December 2020.

[10] Myint MK , Rasmussen C, Thi A, Bustos D, Ringwald P, Lin K. Therapeutic efficacy and artemisinin resistance in northern Myanmar: evidence from in vivo and molecular marker studies. Malar J 2017; 16:143. 28388902 10.1186/s12936-017-1775-2 PMC5383981

[11] Ricotta E , Kwan J. Artemisinin-resistant malaria as a global catastrophic biological threat. Curr Top Microbiol Immunol 2019; 424:33–57. 31218504 10.1007/82_2019_163

[12] von Seidlein L , Peto TJ, Tripura R, et al Novel approaches to control malaria in forested areas of Southeast Asia. Trends Parasitol 2019; 35:388–98. 31076353 10.1016/j.pt.2019.03.011

[13] Hannah K , Ricotta E, Olapeju B, Choiriyyah I. Insecticide-treated nets (ITN) access and use report. 2016. https://www.vector-works.org/resources/itn-access-and-use/. Accessed 14 July 2020.

[14] Lover AA , Sutton BA, Asy AJ, Wilder-Smith A. An exploratory study of treated-bed nets in Timor-Leste: patterns of intended and alternative usage. Malar J 2011; 10:199. 21777415 10.1186/1475-2875-10-199 PMC3155971

[15] Silver JB. Mosquito ecology—field sampling methods. 3rd ed. Springer Netherlands, 2008. doi:10.1007/978-1-4020-6666-5

[16] Schapira A , Boutsika K. Malaria ecotypes and stratification. Adv Parasitol 2012; 78:97–167. 22520442 10.1016/B978-0-12-394303-3.00001-3

[17] World Health Organization. WHO malaria terminology. 2019. https://www.who.int/malaria/publications/atoz/malaria- terminology/en/ . Accessed 14 July 2020.

[18] Wharton-Smith A , Shafique M. A qualitative study to assess consumer preferences and barriers to use of long lasting insecticidal nets in Myanmar. Research Report. Johns Hopkins Bloomberg School of Public Health Center for Communication Programs/Malaria Consortium. 60 pp. https://www.vector-works.org/wp-content/uploads/Networks-Consumer-Preferences-Study-Myanmar-2014-12-23.pdf. Accessed 15 May 2020.

[19] Gryseels C , Peeters Grietens K, Dierickx S, et al. High mobility and low use of malaria preventive measures among the Jarai male youth along the Cambodia-Vietnam border. Am J Trop Med Hyg 2015; 93:810–8. 26283747 10.4269/ajtmh.15-0259 PMC4596604

[20] von Seidlein L , Ikonomidis K, Mshamu S, et al Affordable house designs to improve health in rural Africa: a field study from northeastern Tanzania. Lancet Planet Heal 2017; 1:e188–99. 10.1016/S2542-5196(17)30078-5 29851640

[21] Pulford J , Hetzel MW, Bryant M, Siba PM, Mueller I. Reported reasons for not using a mosquito net when one is available: a review of the published literature. Malar J 2011; 10:83. 21477376 10.1186/1475-2875-10-83 PMC3080352

[22] Hay SI , Sinka ME, Okara RM, et al Developing global maps of the dominant *Anopheles* vectors of human malaria. PLoS Med 2010; 7:e1000209. 20161718 10.1371/journal.pmed.1000209 PMC2817710

[23] Sinka ME , Bangs MJ, Manguin S, et al. The dominant *Anopheles* vectors of human malaria in the Asia-Pacific region: occurrence data, distribution maps and bionomic précis. Parasit Vectors 2011; 4:252. 10.1186/1756-3305-4-89 PMC3127851 21612587

[24] Guyant P , Canavati SE, Chea N, et al Malaria and the mobile and migrant population in Cambodia: a population movement framework to inform strategies for malaria control and elimination. Malar J 2015; 14:83. 26088924 10.1186/s12936-015-0773-5 PMC4474346

[25] World Health Organization. Expert consultation on *Plasmodium knowlesi* malaria to guide malaria elimination strategies. 2017. https://apps.who.int/iris/handle/10665/259130?mode=simple. Accessed 20 June 2020.

[26] William T , Rahman HA, Jelip J, et al. Increasing incidence of *Plasmodium knowlesi* malaria following control of *P. falciparum* and *P. vivax* malaria in Sabah, Malaysia. PLoS Negl Trop Dis 2013; 7:e2026. 23359830 10.1371/journal.pntd.0002026 PMC3554533

[27] William T , Jelip J, Menon J, et al. Changing epidemiology of malaria in Sabah, Malaysia: increasing incidence of *Plasmodium knowlesi*. Malar J 2014; 13:390. 25272973 10.1186/1475-2875-13-390 PMC4195888

[28] Vythilingam I , Hii J. Simian malaria parasites: special emphasis on *Plasmodium knowlesi* and their Anopheles vectors in Southeast Asia. In Anopheles mosquitoes – New insights into malaria vectors (Manguin S. Editor. Rijeka: Intech; 2013: 487-510. https://www.intechopen.com/books/anopheles-mosquitoes-new-insights-into-malaria-vectors/simian-malaria-parasites-special-emphasis-on-plasmodium-knowlesi-and-their-anopheles-vectors-in-sout. Accessed May 10, 2020.

[29] Moyes CL , Shearer FM, Huang Z, et al. Predicting the geographical distributions of the macaque hosts and mosquito vectors of *Plasmodium knowlesi* malaria in forested and non-forested areas. Parasit Vectors 2016; 9:242. 27125995 10.1186/s13071-016-1527-0 PMC4850754

[30] Grigg MJ , Cox J, William T, et al Individual-level factors associated with the risk of acquiring human *Plasmodium knowlesi* malaria in Malaysia: a case-control study. Lancet Planet Heal 2017; 1:97–104. 10.1016/S2542-5196(17)30031-1 PMC5531251 28758162

[31] Hii J , Vythilingam I, Roca-Feltrer A. Human and simian malaria in the Greater Mekong Subregion and challenges for elimination. In Towards Malaria Elimination. Sylvie Manguin and Vas Dev, Editors. IntechOpen; 2018: 95-127. https://www.intechopen.com/books/towards-malaria-elimination-a-leap-forward/human-and-simian-malaria-in-the-greater-mekong-subregion-and-challenges-for-elimination. Accessed May 10, 2020.

[32] Fornace KM , Brock PM, Abidin TR, et al Environmental risk factors and exposure to the zoonotic malaria parasite *Plasmodium knowlesi* across northern Sabah, Malaysia: a population-based cross-sectional survey. Lancet Planet Heal 2019; 3:179–86. 10.1016/S2542-5196(19)30045-2 PMC6484808 31029229

[33] Liberati A , Altman DG, Tetzlaff J, et al The PRISMA statement for reporting systematic reviews and meta-analyses of studies that evaluate health care interventions: explanation and elaboration. J Clin Epidemiol 2009; 339:2700. 10.1016/j.jclinepi.2009.06.006 19631507

[34] Monroe A , Moore S, Koenker H, Lynch M, Ricotta E. Measuring and characterizing night time human behaviour as it relates to residual malaria transmission in sub-Saharan Africa: a review of the published literature. Malar J 2019; 18:6. 30634963 10.1186/s12936-019-2638-9 PMC6329148

[35] Suwonkerd W , Ritthison W, Chung Thuy Ngo, Tainchum K, Bangs MJ, Chareonviriyaphap T. Vector Biology and Malaria Transmission in Southeast Asia. In Anopheles mosquitoes – New insights into malaria vectors (Manguin S. Editor. Rijeka: Intech Open; 2013: 273-325.

[36] Burkot TR , Farlow R, Min M, Espino E, Mnzava A, Russell TL. A global analysis of National Malaria Control Programme vector surveillance by elimination and control status in 2018. Malar J 2019; 18:399. 31801543 10.1186/s12936-019-3041-2 PMC6894334

[37] Hii J , Rueda LM. Malaria vectors in the Greater Mekong Subregion: overview of malaria vectors and remaining challenges. Southeast Asian J Trop Med Public Health 2013; 44(suppl 1):73–165; discussion 306–7. 24159831

[38] Nofal SD , Peto TJ, Adhikari B, et al. How can interventions that target forest-goers be tailored to accelerate malaria elimination in the Greater Mekong Subregion? a systematic review of the qualitative literature. Malar J 2019; 18:32. 30709399 10.1186/s12936-019-2666-5 PMC6359845

[39] Gryseels C , Durnez L, Gerrets R, et al. Re-imagining malaria: heterogeneity of human and mosquito behaviour in relation to residual malaria transmission in Cambodia. Malar J 2015; 14:165. 25908498 10.1186/s12936-015-0689-0 PMC4408599

[40] Durnez L , Mao S, Denis L, Roelants P, Sochantha T, Coosemans M. Outdoor malaria transmission in forested villages of Cambodia. Malar J 2013; 12:329. 24044424 10.1186/1475-2875-12-329 PMC3848552

[41] Bannister-Tyrrell M , Krit M, Sluydts V, et al. Households or hotspots? defining intervention targets for malaria elimination in Ratanakiri Province, Eastern Cambodia. J Infect Dis 2019; 220:1034–43. 31028393 10.1093/infdis/jiz211 PMC6688056

[42] Tangena JA , Thammavong P, Lindsay SW, Brey PT. Risk of exposure to potential vector mosquitoes for rural workers in Northern Lao PDR. PLoS Negl Trop Dis 2017; 11:e0005802. 28742854 10.1371/journal.pntd.0005802 PMC5544251

[43] Population Services International. Vietnam worksite research report. 2017. https://www.psi.org/publication/psi-vietnam-worksite-research-report/. Accessed 23 June 2020.

[44] Edwards HM , Sriwichai P, Kirabittir K, Prachumsri J, Chavez IF, Hii J. Transmission risk beyond the village: entomological and human factors contributing to residual malaria transmission in an area approaching malaria elimination on the Thailand-Myanmar border. Malar J 2019; 18:221. 31262309 10.1186/s12936-019-2852-5 PMC6604376

[45] Edwards HM , Chinh VD, Le Duy B, et al. Characterising residual malaria transmission in forested areas with low coverage of core vector control in central Viet Nam. Parasit Vectors 2019; 12:454. 31533794 10.1186/s13071-019-3695-1 PMC6751671

[46] Smithuis FM , Kyaw MK, Phe UO, et al. Entomological determinants of insecticide-treated bed net effectiveness in Western Myanmar. Malar J 2013; 12:364. 24119994 10.1186/1475-2875-12-364 PMC4015723

[47] Barber BE , William T, Grigg MJ, et al. A prospective comparative study of knowlesi, falciparum, and vivax malaria in Sabah, Malaysia: high proportion with severe disease from *Plasmodium knowlesi* and *Plasmodium vivax* but no mortality with early referral and artesunate therapy. Clin Infect Dis 2013; 56:383–97. 23087389 10.1093/cid/cis902

[48] Chua TH , Manin BO, Vythilingam I, Fornace K, Drakeley CJ. Effect of different habitat types on abundance and biting times of *Anopheles balabacensis* Baisas (Diptera: Culicidae) in Kudat district of Sabah, Malaysia. Parasit Vectors 2019; 12:364. 31345256 10.1186/s13071-019-3627-0 PMC6659233

[49] Rohani A , Fakhriy HA, Suzilah I, et al. Indoor and outdoor residual spraying of a novel formulation of deltamethrin K-Othrine® (Polyzone) for the control of simian malaria in Sabah, Malaysia. PLoS One 2020; 15:e0230860. 32413033 10.1371/journal.pone.0230860 PMC7228059

[50] Rodriguez-Rodriguez D , Maraga S, Lorry L, et al. Repeated mosquito net distributions, improved treatment, and trends in malaria cases in sentinel health facilities in Papua New Guinea. Malar J 2019; 18:364. 31718659 10.1186/s12936-019-2993-6 PMC6852945

[51] Rodriguez D. Epidemiology and control of malaria in Papua New Guinea: from small-scale heterogeneity to large-scale surveillance and targeted response. University of Basel, 2019. https://edoc.unibas.ch/75351. Accessed 23 July 2020.

[52] Kattenberg JH , Gumal DL, Ome-Kaius M, et al. The epidemiology of *Plasmodium falciparum* and *Plasmodium vivax* in East Sepik Province, Papua New Guinea, pre- and post-implementation of national malaria control efforts. Malar J 2020; 19:198. 32503607 10.1186/s12936-020-03265-x PMC7275396

[53] Pollard EJM , MacLaren D, Russell TL, Burkot TR. Protecting the peri-domestic environment: the challenge for eliminating residual malaria. Sci Rep 2020; 10:7018. 32341476 10.1038/s41598-020-63994-6 PMC7184721

[54] World Health Organization. World malaria report 2015—summary. 2015. https://www.who.int/malaria/publications/world-malaria-report-2015/en/. Accessed 24 June 2020.

[55] Koenker H , Kilian A. Recalculating the net use gap: a multi-country comparison of ITN use versus ITN access. PLoS One 2014; 9:e97496. 24848768 10.1371/journal.pone.0097496 PMC4030003

[56] Hetzel MW , Gideon G, Lote N, Makita L, Siba PM, Mueller I. Ownership and usage of mosquito nets after four years of large-scale free distribution in Papua New Guinea. Malar J 2012; 11:192. 22682111 10.1186/1475-2875-11-192 PMC3422192

[57] Eisele TP , Keating J, Littrell M, Larsen D, Macintyre K. Assessment of insecticide-treated bednet use among children and pregnant women across 15 countries using standardized national surveys. Am J Trop Med Hyg 2009; 80:209–14. 19190215

[58] Gryseels C , Bannister-Tyrrell M, Uk S, et al. A critical enquiry into variability of insecticidal net use in Cambodia: implications for assessing appropriateness of malaria elimination interventions. Am J Trop Med Hyg 2019; 100:1424–32. 30994087 10.4269/ajtmh.18-0730 PMC6553892

[59] Hewitt S. Malaria vector control in the Greater Mekong Sub-region: an independent situation analysis and suggestions for improvement. 2018. http://www.vbdc-consulting.com/files/180920.pdf. Accessed 16 June 2020.

[60] Nene L . Bed-net usage determinants and user preference in Cambodia. A user-centered design approach to understanding determinants and preferences associated with bed-net product attributes. Population Services International. PSK. USAID; 2016. Unpublished report, 262 pp.

[61] Incardona S , Vong S, Chiv L, et al. Large-scale malaria survey in Cambodia: novel insights on species distribution and risk factors. Malar J 2007; 6:37. 17389041 10.1186/1475-2875-6-37 PMC1847522

[62] Durnez L , Pareyn M, Mean V, et al. Identification and characterization of areas of high and low risk for asymptomatic malaria infections at sub-village level in Ratanakiri, Cambodia. Malar J 2018; 17:27. 29334956 10.1186/s12936-017-2169-1 PMC5769347

[63] Vythilingam I , Phetsouvanh R, Keokenchanh K, et al. The prevalence of *Anopheles* (Diptera: Culicidae) mosquitoes in Sekong Province, Lao PDR in relation to malaria transmission. Trop Med Int Health 2003; 8:525–35. 12791058 10.1046/j.1365-3156.2003.01052.x

[64] Vythilingam I , Luz BM, Hanni R, Beng TS, Huat TC. Laboratory and field evaluation of the insect growth regulator pyriproxyfen (Sumilarv 0.5G) against dengue vectors. J Am Mosq Control Assoc 2005; 21:296–300. 16252520 10.2987/8756-971X(2005)21[296:LAFEOT]2.0.CO;2

[65] Nonaka D , Laimanivong S, Kobayashi J, et al. Is staying overnight in a farming hut a risk factor for malaria infection in a setting with insecticide-treated bed nets in rural Laos? Malar J 2010; 9:372. 21176242 10.1186/1475-2875-9-372 PMC3224235

[66] Thanh PV , Van Hong N, Van Van N, et al. Epidemiology of forest malaria in Central Vietnam: the hidden parasite reservoir. Malar J 2015; 14:86. 25880664 10.1186/s12936-015-0601-y PMC4342195

[67] Son DH , Thuy-Nhien N, von Seidlein L, et al. The prevalence, incidence and prevention of *Plasmodium falciparum* infections in forest rangers in Bu Gia Map National Park, Binh Phuoc province, Vietnam: a pilot study. Malar J 2017; 16:444. 29110709 10.1186/s12936-017-2091-6 PMC5674731

[68] Ngo CT , Dubois G, Sinou V, et al. Diversity of *Anopheles* mosquitoes in Binh Phuoc and Dak Nong Provinces of Vietnam and their relation to disease. Parasit Vectors 2014; 7:316. 25008314 10.1186/1756-3305-7-316 PMC4227083

[69] Trung HD , Van Bortel W, Sochantha T, Keokenchanh K, Briët OJ, Coosemans M. Behavioural heterogeneity of *Anopheles* species in ecologically different localities in Southeast Asia: a challenge for vector control. Trop Med Int Health 2005; 10:251–62. 15730510 10.1111/j.1365-3156.2004.01378.x

[70] Erhart A , Ngo DT, Phan VK, et al. Epidemiology of forest malaria in central Vietnam: a large scale cross-sectional survey. Malar J 2005; 4:58. 16336671 10.1186/1475-2875-4-58 PMC1325238

[71] Van Bortel W , Trung HD, Hoi LX, et al Malaria transmission and vector behaviour in a forested malaria focus in central Vietnam and the implications for vector control. Malaria J 2010; 9:373. 10.1186/1475-2875-9-373 PMC3224380 21182774

[72] Peeters Grietens K , Xuan XN, Van Bortel W, et al. Low perception of malaria risk among the Ra-glai ethnic minority in south-central Vietnam: implications for forest malaria control. Malar J 2010; 9:23. 20089152 10.1186/1475-2875-9-23 PMC2823606

[73] Grietens KP , Xuan XN, Ribera J, et al. Social determinants of long lasting insecticidal hammock use among the Ra-glai ethnic minority in Vietnam: implications for forest malaria control. PLoS One 2012; 7:e29991. 22253852 10.1371/journal.pone.0029991 PMC3257264

[74] Sanh NH , Dung NV, Thanh NX, Trung TN, Co TV, Cooper RD. Short report: forest malaria in central Vietnam. Am J Trop Med Hyg 2008; 79:652–4. 18981498

[75] Smithuis FM , Kyaw MK, Phe UO, et al. The effect of insecticide-treated bed nets on the incidence and prevalence of malaria in children in an area of unstable seasonal transmission in western Myanmar. Malar J 2013; 12:363. 24119916 10.1186/1475-2875-12-363 PMC3854704

[76] Manin BO , Ferguson HM, Vythilingam I, et al. Investigating the contribution of peri-domestic transmission to risk of zoonotic malaria infection in humans. PLoS Negl Trop Dis 2016; 10:e0005064. 27741235 10.1371/journal.pntd.0005064 PMC5065189

[77] Hetzel MW , Reimer LJ, Gideon G, et al. Changes in malaria burden and transmission in sentinel sites after the roll-out of long-lasting insecticidal nets in Papua New Guinea. Parasit Vectors 2016; 9:340. 27301964 10.1186/s13071-016-1635-x PMC4908799

[78] Reimer LJ , Thomsen EK, Koimbu G, et al. Malaria transmission dynamics surrounding the first nationwide long-lasting insecticidal net distribution in Papua New Guinea. Malar J 2016; 15:25. 26753618 10.1186/s12936-015-1067-7 PMC4709896

[79] Reimer LJ , Thomsen EK, Tisch DJ, et al. Insecticidal bed nets and filariasis transmission in Papua New Guinea. N Engl J Med 2013; 369:745–53. 23964936 10.1056/NEJMoa1207594 PMC3835352

[80] Russell TL , Beebe NW, Bugoro H, et al Frequent blood feeding enables insecticide-treated nets to reduce transmission by mosquitoes that bite predominately outdoors. Malaria J 2016; 15: 156. 10.1186/s12936-016-1195-8 PMC4788858 26969430

[81] Russell TL , Beebe NW, Bugoro H, et al *Anopheles farauti* is a homogeneous population that blood feeds early and outdoors in the Solomon Islands. Malaria J 2016; 15:151. 10.1186/s12936-016-1194-9 PMC4784415 26960327

[82] Russell TL , Beebe NW, Bugoro H, et al Determinants of host feeding success by *Anopheles farauti*. Malaria J 2016; 15:152. 10.1186/s12936-016-1168-y PMC4785651 26964528

[83] Solomon Islands National Statistics Office. Demographic and health survey 2015. 2015. http://sdd.spc.int/media/155. Accessed 6 July 2020.

[84] Somboon P , Lines J, Aramrattana A, Chitprarop U, Prajakwong S, Khamboonruang C. Entomological evaluation of community-wide use of lambdacyhalothrin-impregnated bed nets against malaria in a border area of north-west Thailand. Trans R Soc Trop Med Hyg 1995; 89:248–54. 7660424 10.1016/0035-9203(95)90525-1

[85] Somboon P , Aramrattana A, Lines J, Webber R. Entomological and epidemiological investigations of malaria transmission in relation to population movements in forest areas of north-west Thailand. Southeast Asian J Trop Med Public Health 1998; 29:3–9. 9740259

[86] Hetzel MW , Choudhury AA, Pulford J, et al. Progress in mosquito net coverage in Papua New Guinea. Malar J 2014; 13:242. 24961245 10.1186/1475-2875-13-242 PMC4077150

[87] World Health Organization. Countries of the Greater Mekong are stepping up to end malaria. 2018. https://www.who.int/malaria/publications/atoz/greater-mekong-bulletin-7/en/. Accessed 6 July 2020.

[88] Kheang ST , Lin MA, Lwin S, et al Malaria case detection among mobile populations and migrant workers in Myanmar: comparison of 3 service delivery approaches. Glob Heal Sci Pra ct 2018; 6:381–6. 10.9745/GHSP-D-17-00318 PMC6024619 29875157

[89] Canavati SE , Lawpoolsri S, Quintero CE, et al Village malaria worker performance key to the elimination of artemisinin-resistant malaria: a western Cambodia health system assessment. Malar J 2016; 15. 10.1186/s12936-016-1322-6 PMC4875644 27206729

[90] Rosenberg R , Andre RG, Somchit L. Highly efficient dry season transmission of malaria in Thailand. Trans R Soc Trop Med Hyg 1990; 84:22–8. 2189240 10.1016/0035-9203(90)90367-n

[91] Green CA , Rattanarithikul R, Pongparit S, Sawadwongporn P, Baimai V. A newly-recognized vector of human malarial parasites in the oriental region, *Anopheles* (*Cellia*) *pseudowillmori* (Theobald, 1910). Trans R Soc Trop Med Hyg 1991; 85:35–6. 2068752 10.1016/0035-9203(91)90143-m

[92] Harbach RE , Gingrich JB, Pang LW. Some entomological observations on malaria transmission in a remote village in northwestern Thailand. J Am Mosq Control Assoc 1987; 3:296–301. 3333058

[93] Prasittisuk C , Prasittisuk M, Phatipongse S. Ketrangsee S, Krachailin S, Aumaung B, Chaiprasittigul P, Lucchini A, Ngarmathom S. Epidemiological aspects of malaria transmission in eastern Thailand. Proc Third Malar Res Conf 1989. Thailand, 18-20 October, pp 22-23.

[94] Somboon P , Aramrattana A, Lines J, Webber R. Entomological and epidemiological investigations of malaria transmission in relation to population movements in forest areas of north-west Thailand. Southeast Asian J Trop Med Public Health 1998; 29:3–9. 9740259

[95] Tangena JA , Thammavong P, Wilson AL, Brey PT, Lindsay SW. Risk and control of mosquito-borne diseases in Southeast Asian rubber plantations. Trends Parasitol 2016; 32:402–15. 26907494 10.1016/j.pt.2016.01.009

[96] National Institute of Public Health. Report of the Cambodia National Malaria Baseline Survey 2004. 2005. https://www.malariaconsortium.org/media-downloads/171. Accessed 6 July 2020.

[97] National Institute of Public Health. Cambodia Malaria Survey 2007 report. 2007. https://www.malariasurveys.org/documents/CMS2007 report_22Sep10_FINAL.pdf. Accessed 6 July 2020.

[98] National Centre for Parasitology Entomology and Malaria Control. Cambodia Malaria Survey 2010. 2010. https://malariasurveys.org/documents/CMS 2010 GF Report (FINAL).pdf. Accessed 6 July 2020.

[99] National Centre for Parasitology Entomology and Malaria Control. Cambodia Malaria Survey 2013. 2013. https://www.malariaconsortium.org/media-downloads/624/Cambodia Malaria Survey 2013. Accessed 6 July 2020.

[100] St Laurent B , Oy K, Miller B, et al. Cow-baited tents are highly effective in sampling diverse *Anopheles* malaria vectors in Cambodia. Malar J 2016; 15:440. 27577697 10.1186/s12936-016-1488-y PMC5004278

[101] Wong ML , Chua TH, Leong CS, et al. Seasonal and spatial dynamics of the primary vector of *Plasmodium knowlesi* within a major transmission focus in Sabah, Malaysia. PLoS Negl Trop Dis 2015; 9:e0004135. 26448052 10.1371/journal.pntd.0004135 PMC4598189

[102] Peeters Grietens K , Gryseels C, Verschraegen G. Misdirection in the margins of malaria elimination methods. Critical Pub Hlth 2019; 29: 390- 400.

[103] Atkinson JA , Bobogare A, Vallely A, et al A cluster randomized controlled cross-over bed net acceptability and preference trial in Solomon Islands: community participation in shaping policy for malaria elimination. Malar J 2009; 8:298. 20015402 10.1186/1475-2875-8-298 PMC2803192

[104] Atkinson JA , Fitzgerald L, Toaliu H, et al. Community participation for malaria elimination in Tafea Province, Vanuatu. Part I. Maintaining motivation for prevention practices in the context of disappearing disease. Malar J 2010; 9:93. 20380748 10.1186/1475-2875-9-93 PMC2873527

[105] Whidden CE , Premaratne RG, Jayanetti SR, Fernando SD. Patterns and predictive factors of long-lasting insecticidal net usage in a previously high malaria endemic area in Sri Lanka: a cross-sectional survey. Trans R Soc Trop Med Hyg 2015; 109:553–62. 26187622 10.1093/trstmh/trv056

[106] Atkinson JA , Bobogare A, Fitzgerald L, et al A qualitative study on the acceptability and preference of three types of long-lasting insecticide-treated bed nets in Solomon Islands: implications for malaria elimination. Malar J 2009; 8:119. 19497127 10.1186/1475-2875-8-119 PMC2699345

[107] Koenker H , Yukich JO. Effect of user preferences on ITN use: a review of literature and data. Malar J 2017; 16:233. 28571583 10.1186/s12936-017-1879-8 PMC5455118

[108] Rollback Malaria Partnership, Global Fund. Malaria Matchbox tool: an equity assessment tool to improve the effectiveness of malaria programs. 2020. https://endmalaria.org/sites/default/files/Malaria Matchbox Tool_en_web.pdf. Accessed 6 July 2020.

[109] Wangdi K , Clements AC. Ending malaria transmission in the Asia Pacific Malaria Elimination Network (APMEN) countries: challenges and the way forward. In Towards Malaria Elimination - A Leap Forward. Sylvie Manguin and Vas Dev, Editors. IntechOpen; 2018: 201–32. https://cdn.intechopen.com/pdfs/59967.pdf

[110] Vinit RJ , Timinao L, Bubun N, et al Decreased bioefficacy of long-lasting insecticidal nets and the resurgence of malaria in Papua New Guinea. Nat Commun 2020; 11:3646. 32686679 10.1038/s41467-020-17456-2 PMC7371689

[111] Killeen GF , Kihonda J, Lyimo E, et al. Quantifying behavioural interactions between humans and mosquitoes: evaluating the protective efficacy of insecticidal nets against malaria transmission in rural Tanzania. BMC Infect Dis 2006; 6:161. 17096840 10.1186/1471-2334-6-161 PMC1657018

[112] Vythilingam et al. Epidemiology of malaria in Attapeu Province, Lao. PDR in relation to entomological parameters. Trans R Soc Trop Med Hyg 2005; 99:833–39. 16112154 10.1016/j.trstmh.2005.06.012

[113] Ismail IA , Notananda V, Schepens J. Studies on malaria and responses of *Anopheles balabacensis balabacensis* and *Anopheles minimus* to DDT residual spraying in Thailand. Acta Trop 1975; 32:206–31. 1984

[114] Suwonkerd W , Amg-Ung B, Rimwangtrakul K, et al A field study on the response of *Anopheles dirus* to DDT and fenitrothion sprayed to huts in Phetchabun province Thailand. Trop Med 1990; 32:1–5.

[115] Nustsathapana S , Sawasdiwongphorn P, Chitprarop U, Cullen JR. The behaviour of *Anopheles minimus* Theobald (Diptera: Culicidae) subjected to differing levels of DDT selection pressure in northern Thailand. Bull Entomol Res 1986; 76:303–12.

[116] Malaithong N , Polsomboon S, Poolprasert P, et al. Human-landing patterns of *Anopheles dirus* sensu lato (Diptera: Culicidae) in experimental huts treated with DDT or deltamethrin. J Med Entomol 2010; 47:823–32. 20939377 10.1603/me09016

[117] Pleass RJ , Armstrong JRM, Curtis CF, Jawara M, Lindsay SW. Comparison of permethrin treatments for bednets in The Gambia. Bull Entomol Res 1993; 83:133–9.

[118] Pimnon S , Bhumiratana A. Adaptation of anopheles vectors to anthropogenic malaria-associated rubber plantations and indoor residual spraying: establishing population dynamics and insecticide susceptibility. Can J Infect Dis Med Microbiol 2018; 2018:9853409. 30034563 10.1155/2018/9853409 PMC6032653

[119] Malaria Elimination Initiative. Entomological Surveillance Planning Tool. 2020. UCSF Global Health Group’s Malaria Elimination Initiative (MEI). Institute for Global Health Sciences. http://www.shrinkingthemalariamap.org/entomological-surveillance-planning-tool-espt. Accessed 27 October 2020.

[120] Monroe A , Moore S, Okumu F, et al. Methods and indicators for measuring patterns of human exposure to malaria vectors. Malar J 2020; 19:207. 32546166 10.1186/s12936-020-03271-z PMC7296719

[121] Sanann N , Peto TJ, Tripura R, et al. Forest work and its implications for malaria elimination: a qualitative study. Malar J 2019; 18:376. 31771587 10.1186/s12936-019-3008-3 PMC6880349

[122] Xu JW , Liu H, Zhang Y, Guo XR, Wang JZ. Risk factors for border malaria in a malaria elimination setting: a retrospective case-control study in Yunnan, China. Am J Trop Med Hyg 2015; 92. 10.4269/ajtmh.14-0321 PMC4350546 25601994

[123] Lyttleton C . Deviance and resistance: malaria elimination in the Greater Mekong Subregion. Soc Sci Med 2016; 150:144–52. 26751710 10.1016/j.socscimed.2015.12.033

[124] Parker DM , Carrara VI, Pukrittayakamee S, McGready R, Nosten FH. Malaria ecology along the Thailand-Myanmar border. Malar J 2015; 14:388. 26437860 10.1186/s12936-015-0921-y PMC4594738

[125] Shoklo Malaria Research Unit. Malaria elimination task force interim report. 2016. https://www.shoklo-unit.com/sites/default/files/reports/malaria-elimination-task-force/metf_interim_report_feb2016.pdf. Accessed 16 June 2020.

[126] Grietens KP , Xuan XN, Ribera J, et al. Social determinants of long lasting insecticidal hammock use among the Ra-glai ethnic minority in Vietnam: implications for forest malaria control. PLoS One 2012; 7:e29991. 22253852 10.1371/journal.pone.0029991 PMC3257264

[127] Ipa M , Widawati M, Laksono AD, Kusrini I, Dhewantara PW. Variation of preventive practices and its association with malaria infection in eastern Indonesia: findings from community-based survey. PLoS One 2020; 15:e0232909. 32379812 10.1371/journal.pone.0232909 PMC7205284

[128] Ranson H , Lissenden N. Insecticide resistance in African *Anopheles* Mosquitoes: a worsening situation that needs urgent action to maintain malaria control. Trends Parasitol 2016; 32:187–96. 26826784 10.1016/j.pt.2015.11.010

[129] World Health Organization. Global report on insecticide resistance in malaria vectors: 2010–2016. 2018. https://www.who.int/malaria/publications/atoz/9789241514057/en/. Accessed 24 June 2020.

[130] Chareonviriyaphap T , Bangs MJ, Suwonkerd W, Kongmee M, Corbel V, Ngoen-Klan R. Review of insecticide resistance and behavioral avoidance of vectors of human diseases in Thailand. Parasit Vectors 2013; 6:280. 24294938 10.1186/1756-3305-6-280 PMC3850650

[131] World Health Organization. Global plan for insecticide resistance management in malaria vectors. 2012. https://www.who.int/malaria/publications/atoz/gpirm/en/. Accessed 16 June 2020.

[132] Charlwood JD , Graves PM. The effect of permethrin-impregnated bednets on a population of *Anopheles farauti* in coastal Papua New Guinea. Med Vet Entomol 1987; 1:319–27. 2979548 10.1111/j.1365-2915.1987.tb00361.x

[133] Taylor B . Changes in the feeding behaviour of a malaria vector, *Anopheles farauti Lav.*, following use of DDT as a residual spray in houses in the British Solomon Islands Protectorate. Trans R Entomol Soc London 1975; 127:277–92.

[134] Thomsen E , Koimbu G, Pulford J. Mosquito behaviour change after distribution of bednets results in decreased protection against malaria exposure. J Infect Dis 2016; 215:790–7. 10.1093/infdis/jiw615 PMC5388271 28007921

[135] Roberts DR , Alecrim WD, Hshieh P, et al. A probability model of vector behavior: effects of DDT repellency, irritancy, and toxicity in malaria control. J Vector Ecol 2000; 25:48–61. 10925797

[136] Miller JR , Siegert PY, Amimo FA, Walker ED. Designation of chemicals in terms of the locomotor responses they elicit from insects: an update of Dethier *et al*. (1960). J Econ Entomol 2009; 102:2056–60. 20069831 10.1603/029.102.0606

[137] Grieco JP , Achee NL, Chareonviriyaphap T, et al A new classification system for the actions of IRS chemicals traditionally used for malaria control. PLoS One 2007; 2:716. 10.1371/journal.pone.0000716 PMC1934935 17684562

[138] Govella NJ , Chaki PP, Killeen GF. Entomological surveillance of behavioural resilience and resistance in residual malaria vector populations. Malar J 2013; 12:124. 23577656 10.1186/1475-2875-12-124 PMC3637503

[139] Bugoro H , Cooper RD, Butafa C, et al Bionomics of the malaria vector *Anopheles farauti* in Temotu Province, Solomon Islands: issues for malaria elimination. Malar J 2011; 10. 10.1186/1475-2875-10-133 PMC3123245 21592366

[140] Keven JB , Henry-Halldin CN, Thomsen EK, et al Short report: pyrethroid susceptibility in natural populations of the *Anopheles punctulatus* group (Diptera: Culicidae) in Papua New Guinea. Am J Trop Med Hyg 2010; 83:1259–61. 21118931 10.4269/ajtmh.2010.10-0422 PMC2990041

[141] Henry-Halldin CN , Nadesakumaran K, Keven JB, et al. Multiplex assay for species identification and monitoring of insecticide resistance in *Anopheles punctulatus* group populations of Papua New Guinea. Am J Trop Med Hyg 2012; 86:140–51. 22232465 10.4269/ajtmh.2012.11-0503 PMC3247123

[142] Koimbu G , Czeher C, Katusele M, et al. Status of insecticide resistance in Papua New Guinea: an update from nation-wide monitoring of anopheles mosquitoes. Am J Trop Med Hyg 2018; 98:162–5. 29141726 10.4269/ajtmh.17-0454 PMC5928719

[143] Quiñones ML , Norris DE, Conn JE, et al. Insecticide resistance in areas under investigation by the International Centers of Excellence for Malaria Research: a challenge for malaria control and elimination. Am J Trop Med Hyg 2015; 93:69–78. 26259947 10.4269/ajtmh.14-0844 PMC4574276

[144] Sumarnrote A , Overgaard HJ, Marasri N, et al. Status of insecticide resistance in *Anopheles* mosquitoes in Ubon Ratchathani province, Northeastern Thailand. Malar J 2017; 16:299. 28743278 10.1186/s12936-017-1948-z PMC5526291

[145] Van Bortel W , Trung HD, Thuan LK, et al The insecticide resistance status of malaria vectors in the Mekong region. Malar J 2008; 7. 10.1186/1475-2875-7-102 PMC2467428 18534006

[146] Marcombe S , Chonephetsarath S, Thammavong P, Brey PT. Alternative insecticides for larval control of the dengue vector *Aedes aegypti* in Lao PDR: insecticide resistance and semi-field trial study. Parasit Vectors 2018; 11. 10.1186/s13071-018-3187-8 PMC6278129 30509299

[147] Cui F , Raymond M, Qiao CL. Insecticide resistance in vector mosquitoes in China. Pest Manag Sci 2006; 62:1013–22. 16953491 10.1002/ps.1288

[148] Suman DS , Wang Y, Dong L, Gaugler R. Effects of larval habitat substrate on pyriproxyfen efficacy against *Aedes albopictus* (Diptera: Culicidae). J Med Entomol 2013; 50:1261–6. 24843930 10.1603/me13068

[149] Chang X , Zhong D, Fang Q, et al Multiple resistances and complex mechanisms of *Anopheles sinensis* mosquito: a major obstacle to mosquito-borne diseases control and elimination in China. PLoS Negl Trop Dis 2014; 8:2889. 10.1371/journal.pntd.0002889 PMC4031067 24852174

[150] Fang Y , Shi WQ, Wu JT, Li YY, Xue JB, Zhang Y. Resistance to pyrethroid and organophosphate insecticides, and the geographical distribution and polymorphisms of target-site mutations in voltage-gated sodium channel and acetylcholinesterase 1 genes in *Anopheles sinensis* populations in Shanghai, China. Parasit Vectors 2019; 12:396. 31399130 10.1186/s13071-019-3657-7 PMC6688361

[151] Chen S , Qin Q, Zhong D, et al Insecticide resistance status and mechanisms of *Anopheles sinensis* (Diptera: Culicidae) in Wenzhou, an important coastal port city in China. J Med Entomol 2019; 56:803–10. 10.1093/jme/tjz001 PMC6467641 30715428

[152] Pulford J , Kurumop SF, Ura Y, Siba PM, Mueller I, Hetzel MW. Malaria case management in Papua New Guinea following the introduction of a revised treatment protocol. Malar J 2013; 12:433. 24279720 10.1186/1475-2875-12-433 PMC4222867

[153] Burkot TR , Dye C, Graves PM. An analysis of some factors determining the sporozoite rates, human blood indexes, and biting rates of members of the *Anopheles punctulatus* complex in Papua New Guinea. Am J Trop Med Hyg 1989; 40:229–34. 2929848 10.4269/ajtmh.1989.40.229

[154] Charlwood JD , Graves PM, Alpers MP. The ecology of the *Anopheles punctulatus* group of mosquitoes from Papua New Guinea: a review of recent work. P N G Med J 1986; 29:19–26. 3463014

[155] Bockarie MJ , Hii JL, Alexander ND, et al. Mass treatment with ivermectin for filariasis control in Papua New Guinea: impact on mosquito survival. Med Vet Entomol 1999; 13:120–3. 10484157 10.1046/j.1365-2915.1999.00159.x

[156] Pasay CJ , Yakob L, Meredith HR, et al. Treatment of pigs with endectocides as a complementary tool for combating malaria transmission by *Anopheles farauti* (s.s.) in Papua New Guinea. Parasit Vectors 2019; 12:124. 30890165 10.1186/s13071-019-3392-0 PMC6423892

[157] Keven JB , Reimer L, Katusele M, et al. Plasticity of host selection by malaria vectors of Papua New Guinea. Parasit Vectors 2017; 10:95. 28222769 10.1186/s13071-017-2038-3 PMC5320767

[158] Garros C , Marchand RP, Quang NT, Hai NS, Manguin S. First record of *Anopheles minimu*s C and significant decrease of *An. minimus* A in central Vietnam. J Am Mosq Control Assoc 2005; 21:139–43. 16033115 10.2987/8756-971X(2005)21[139:FROAMC]2.0.CO;2

[159] Over M , Bakote’e B, Velayudhan R, Wilikai, Graves PM. Impregnated nets or DDT residual spraying? Field effectiveness of malaria prevention techniques in Solomon Islands. Am J Trop Med Hyg 2004; 71:214–23. 15331840

[160] Dev V , Manguin S. Biology, distribution and control of *Anopheles (Cellia) minimus* in the context of malaria transmission in northeastern India. Parasites Vectors 2016; 9. 10.1186/s13071-016-1878-6 PMC5111344 27846911

[161] Dev V , Sangma BM, Dash AP. Persistent transmission of malaria in Garo hills of Meghalaya bordering Bangladesh, north-east India. Malar J 2010; 9:263. 20858290 10.1186/1475-2875-9-263 PMC2955675

[162] Parajuli M , Shrestha S, Vaidya R, White G. Nation-wide disappearance of *Anopheles minimus* Theobald, 1901, previously the principal malaria vector in Nepal. Trans R Soc Trop Med Hyg 1981; 75.

[163] Terrenato L , Shrestha S, Dixit KA, et al. Decreased malaria morbidity in the Tharu people compared to sympatric populations in Nepal. Ann Trop Med Parasitol 1988; 82:1–11. 3041928 10.1080/00034983.1988.11812202

[164] Harrison BA . Medical entomology studies—XIII. The Myzomyia series of *Anopheles* (*Cellia*) in Thailand, with emphasis on intra-interspecific variations (Diptera: Culicidae). Contrib Am Entomol Inst 1980; 17:1–195.

[165] Garros C , Van Bortel W, Trung HD, Coosemans M, Manguin S. Review of the Minimus complex of *Anopheles,* main malaria vector in Southeast Asia: from taxonomic issues to vector control strategies. Trop Med Int Health 2006; 11:102–14. 16398761 10.1111/j.1365-3156.2005.01536.x

[166] Li ZZ , Zhang MC, Wus YG, Zhong BL, Lin GY, Huang H. Trial of deltamethrin impregnated bed nets for the control of malaria transmitted by *Anopheles sinensis* and *Anopheles anthropophagus*. Am J Trop Med Hyg 1989; 40:356–9. 2712195

[167] Zhang Z , Yang C. Application of deltamethrin-impregnated bednets for mosquito and malaria control in Yunnan, China. Southeast Asian J Trop Med Public Health 1996; 27:367–71. 9280005

[168] Muirhead-Thomson RC . The significance of irritability, behaviouristic avoidance and allied phenomena in malaria eradication. Bull World Health Organ 1960. PMC2555353 14425069

[169] Spencer T , Spencer M, Venters D. Malaria vectors in Papua New Guinea. PNG Med J. 1974; 17:22-30.

[170] Gatton ML , Chitnis N, Churcher T, et al. The importance of mosquito behavioural adaptations to malaria control in Africa. Evolution 2013; 67:1218–30. 23550770 10.1111/evo.12063 PMC3655544

[171] Monroe A , Mihayo K, Okumu F, et al. Human behaviour and residual malaria transmission in Zanzibar: findings from in-depth interviews and direct observation of community events. Malar J 2019; 18:220. 31262306 10.1186/s12936-019-2855-2 PMC6604484

[172] Alout H , Roche B, Dabiré RK, Cohuet A. Consequences of insecticide resistance on malaria transmission. PLoS Pathog 2017; 13:e1006499. 28880906 10.1371/journal.ppat.1006499 PMC5589250

[173] Sukkanon C , Bangs MJ, Nararak J, Hii J, Chareonviriyaphap T. Discriminating lethal concentrations for transfluthrin, a volatile pyrethroid compound for mosquito control in Thailand. J Am Mosq Control Assoc 2019; 35:258–66. 31922934 10.2987/19-6832.1

[174] Masalu JP , Finda M, Killeen GF, Ngowo HS, Pinda PG, Okumu FO. Creating mosquito-free outdoor spaces using transfluthrin-treated chairs and ribbons. Malar J 2020; 19:109. 32156280 10.1186/s12936-020-03180-1 PMC7063784

[175] Sangoro OP , Gavana T, Finda M, et al. Evaluation of personal protection afforded by repellent-treated sandals against mosquito bites in south-eastern Tanzania. Malar J 2020; 19:148. 32268907 10.1186/s12936-020-03215-7 PMC7140554

[176] Kawada H , Nakazawa S, Shimabukuro K, Ohashi K, Kambewa EA, Foster Pemba D. Effect of metofluthrin-impregnated spatial repellent devices combined with new long-lasting insecticidal nets (Olyset® Plus) on pyrethroid-resistant malaria vectors and malaria prevalence: field trial in south-eastern Malawi. Jpn J Infect Dis 2020; 73:124–31. 31666498 10.7883/yoken.JJID.2019.311

[177] Ministry of Health. Cambodia Malaria Elimination Action Framework, 2016–2020. 2016. https://www.cnm.gov.kh/userfiles/Cambodia Malaria Elimination_English FINAL.pdf. Accessed 16 June 2020.

[178] Lin K , Aung S, Lwin S, Min H, Aye NN, Webber R. Promotion of insecticide-treated mosquito nets in Myanmar. Southeast Asian J Trop Med Public Health 2000; 31:444–7. 11288998

[179] Linn SY , Maung TM, Tripathy JP, et al Barriers in distribution, ownership and utilization of insecticide-treated mosquito nets among migrant population in Myanmar, 2016: a mixed methods study. Malar J 2019; 18:172. 31088451 10.1186/s12936-019-2800-4 PMC6518764

[180] Nyunt MH , Aye KM, Kyaw MP, et al. Challenges in universal coverage and utilization of insecticide-treated bed nets in migrant plantation workers in Myanmar. Malar J 2014; 13:211. 24888548 10.1186/1475-2875-13-211 PMC4058704

[181] Garley AE , Ivanovich E, Eckert E, Negroustoueva S, Ye Y. Gender differences in the use of insecticide-treated nets after a universal free distribution campaign in Kano State, Nigeria: post-campaign survey results. Malar J 2013; 12:119. 23574987 10.1186/1475-2875-12-119 PMC3635971

[182] Wen S , Harvard KE, Gueye CS, Canavati S, Chancellor A, et al. Targeting populations at higher risk for malaria: a survey of national malaria elimination programmes in the Asia Pacific. Malar J 2016; 15: 271. 27165296 10.1186/s12936-016-1319-1 PMC4863339

[183] Open Development Cambodia. Forest cover. 2016. https://opendevelopmentcambodia.net/profiles/forest-cover/. Accessed 16 June 2020.

[184] Davis KF , Yu K, Rulli MC, Pichdara L, D’Odorico P. Accelerated deforestation driven by large-scale land acquisitions in Cambodia. Nat Geosci 2015; 8:772–5.

[185] Thongsripong P , Green A, Kittayapong P, Kapan D, Wilcox B, Bennett S. Mosquito vector diversity across habitats in central Thailand endemic for dengue and other arthropod-borne diseases. PLoS Negl Trop Dis 2013; 7:e2507. 24205420 10.1371/journal.pntd.0002507 PMC3814347

[186] Overgaard HJ , Suwonkerd W, Hii J. The Malaria Landscape: Mosquitoes, Transmission, Landscape, Insecticide Resistance, and Integrated Control in Thailand. In: Morand S, Dujardin J, Lefait-Robin R, Apiwathnasorn C, editors. Socio-Ecological Dimensions of Infectious Diseases in Southeast Asia. Singapore: Springer Science and Business Media; 2015. p. 123-53.

[187] Morand S , Dujardin Jean-Pierre, Lefait-Robin R, Apiwathnasorn C. Socio-ecological dimensions of infectious diseases in Southeast Asia. 2015. Springer, Singapore. 338 pp.

[188] Govella NJ , Ferguson H. Why use of interventions targeting outdoor biting mosquitoes will be necessary to achieve malaria elimination. Front Physiol 2012; 3:199. 22701435 10.3389/fphys.2012.00199 PMC3372949

[189] Killeen GF , Marshall JM, Kiware SS, et al. Measuring, manipulating and exploiting behaviours of adult mosquitoes to optimise malaria vector control impact. BMJ Glob Health 2017; 2:e000212. 10.1136/bmjgh-2016-000212 PMC5444085 28589023

[190] Killeen GF , Chaki PP, Reed TE, Moyes CL, Govella NJ. Entomological surveillance as a cornerstone of malaria elimination: a critical appraisal. In Towards Malaria Elimination - A Leap Forward. Sylvie Manguin and Vas Dev, Editors. IntechOpen; 2018: 403-29. https://cdn.intechopen.com/pdfs/61802.pdf, Accessed Oct 27, 2020.

